# Therapeutic effects and mechanisms of N-(9,10-anthraquinone-2-ylcarbonyl) xanthine oxidase inhibitors on hyperuricemia

**DOI:** 10.3389/fphar.2022.950699

**Published:** 2022-09-02

**Authors:** Tianshu Gao, Jin Xu, Yuxiao Xiao, Jiaqi Li, Weifeng Hu, Xiaoyu Su, Xudong Shen, Wan Yu, Zhen Chen, Baosheng Huang, Honglei Li, Xing Wang

**Affiliations:** ^1^ Department of Pharmacology, School of Pharmacy, China Pharmaceutical University, Nanjing, China; ^2^ Department of Nephrology, Jurong Hospital Affiliated to Jiangsu University, Jurong, China; ^3^ School of Basic Medicine and Clinical Pharmacy, China Pharmaceutical University, Nanjing, China; ^4^ Department of Pathology, Jiangsu Province Hospital on Integration of Chinese and Western Medicine, Nanjing, China; ^5^ Department of Neurosurgical, Jiangsu Province Hospital on Integration of Chinese and Western Medicine, Nanjing, China; ^6^ Department of Neurosurgery, Sir Run Run Hospital, Nanjing Medical University, Nanjing, China; ^7^ Department of Pharmacy, Kangda College of Nanjing Medical University, Lianyungang, China

**Keywords:** anthraquinone, hyperuricemia, xanthine oxidase inhibitor, uric acid transporter, mechanism

## Abstract

**Objective:** To observe the antioxidative effects of N-(9,10-anthraquinone-2-ylcarbonyl) xanthine oxidase inhibitors (NAY) *in vitro* and *in vivo* models of hyperuricemia and explore the mechanism.

**Methods:** A classical experimental method of acute toxicity and a chronic toxicity test were used to compare the toxic effects of different doses of NAY in mice. The hyperuricemia mouse model was established by gavage of potassium oxonate *in vivo*. After treatment with different doses of NAY (low dose: 10 mg/kg, medium dose: 20 mg/kg, and high dose: 40 mg/kg) and allopurinol (positive drug, 10 mg/kg), observe the levels of uric acid (UA), creatinine (CRE), and urea nitrogen (BUN) in urine and serum, respectively, and detect the activities of xanthine oxidase in the liver. The hyperuricemia cell model was induced by adenosine and xanthine oxidase *in vitro*. The cells were given different doses of NAY (50, 100, and 200 μmol/L) and allopurinol (100 μmol/L). Then the culture supernatant UA level of the medium was measured. The next step was to detect the xanthine oxidase activity in the liver and AML12 cells, and the levels of tumor necrosis factor-α (TNF-α), interleukin-6 (IL-6), interleukin-1β (IL-1β), and NOD-like receptor thermal protein domain-associated protein 3 (NLRP3) inflammatory factors in the kidney and serum of mice. Western blot was used to detect xanthine oxidase protein expression in mouse liver tissue and AML12 cells, ASC, Caspase-1, NLRP3, GLUT9, OAT1, and OAT3 protein expression in mouse kidney tissue and HK-2 cells. Hematoxylin–eosin staining was used to stain the liver and kidney tissues of mice and observe the tissue lesions.

**Results:** NAY had little effect on blood routine and biochemical indexes of mice, but significantly reduced the serum UA level. NAY significantly reduced the level of UA in hyperuricemia mice and cells by inhibiting xanthine oxidase activity and reduced the levels of TNF-α, IL-6, and other inflammatory factors in serum and kidney of mice. NAY can inhibit inflammation by inhibiting the NLRP3 pathway. In addition, NAY can downregulate GLUT9 protein expression and upregulate OAT1 and OAT3 protein expression to reduce the UA level by promoting UA excretion and inhibiting UA reabsorption.

**Conclusion:** These findings suggested that NAY produced dual hypouricemic actions. On the one hand, it can inhibit the formation of UA by inhibiting xanthine oxidase inhibitors activity, and on the other hand, it can promote the excretion of UA by regulating the UA transporter. It provides new ideas for the development of hyperuricemia drugs in the future.

## 1 Introduction

Hyperuricemia is a metabolic disease caused by abnormal purine metabolism or reduced uric acid (UA) excretion and is mainly characterized by elevated blood UA (Arromdee et al., 2002; Lv et al., 2013). UA is the final product of purine nucleotide metabolism in the human body. It is mainly synthesized and catalyzed by xanthine oxidase (XOD), which is widely distributed in the liver and then excreted by the kidney (Sanchez-Lozada et al., 2008). Studies have shown that long-term hyperuricemia can cause certain damage to blood vessels, heart, liver, and kidney and is closely related to the occurrence and development of metabolic syndrome such as hypertension, hyperlipidemia, obesity, and insulin resistance (Choi and Ford, 2007; Li et al., 2013). Therefore, hyperuricemia has become one of the important diseases, threatening human health. With the improvement of people’s living standard and the change of dietary pattern, the incidence of hyperuricemia is increasing. At present, hyperuricemia is mainly treated by increasing UA excretion or inhibiting XOD activity. The most commonly used drugs are XOD inhibitors such as allopurinol, which reduce UA by inhibiting XOD activity. However, only less than 40% of patients taking allopurinol can reduce serum UA concentration to the target level, and long-term use can cause liver and kidney damage (Madero et al., 2009; Liu et al., 2016), allergy, bone marrow suppression, gastrointestinal reactions, *etc.* (Burns and Wortmann, 2011; Rees et al., 2014), which limits clinical application to a certain extent. Because of the lack of drugs that work similarly, allopurinol has been widely used in the treatment of hyperuricemia for decades. Finding a drug that is better tolerated and comparable to or superior to allopurinol would be an important advance in the treatment of hyperuricemia. In February 2009, the FDA approved febuxostat (Schumacher, 2005) for the treatment of hyperuricemia in the United States, following the European Union’s approval in May 2008. Febuxostat, as a new anti-UA drug, ended the market dominance of allopurinol and created a new era of gout treatment. However, due to the wide price gap between febuxostat and allopurinol, febuxostat is focused on patients with moderate renal insufficiency and those who have failed or are intolerant to allopurinol treatment. Therefore, we need to develop more powerful, less toxic, and cheaper UA-lowering drugs to relieve the pain of gout patients.

In humans, the kidneys play a key role in maintaining circulating UA concentrations, as more than 70% of UA excretion in the body is accomplished through the kidneys (Lipkowitz, 2012). However, renal damage to UA excretion can lead to hyperuricemia. Renal excretion of UA is accomplished by two types of UA transporters: UA reabsorption transporter and UA excretion transporter. GLUT9 is an important urate exchanger protein in the renal proximal tubule. Its dysfunction causes renal hypoexcretion type hyperuricemia (Maiuolo et al., 2016). There are OAT1 and OAT3 on the basolateral membrane of proximal tubule epithelial cells, and these two proteins can transport urate from the blood to epithelial cells [29]. Inhibition of the reabsorptive effects of OAT1 and OAT3 may alleviate hyperuricemia (Ichida et al., 2012). Therefore, promotion of uric acid excretion through modulation of these urate transporters remains an attractive therapeutic target for hyperuricemia.

Structure–activity relationship (SAR) analysis and molecular modeling studies demonstrated that the 9,10-anthraquinone moiety located at the outer region of XO active pocket and performed a role of the lipophilic fragment as in the case of the isobutoxy group of febuxostat (Zhang et al., 2017). We synthesized 9,10-anthraquinone compounds with XOD inhibitor activity, after previous experiments (Zhang et al., 2018) demonstrating that NAY acts best.

**FIGURE 1:**
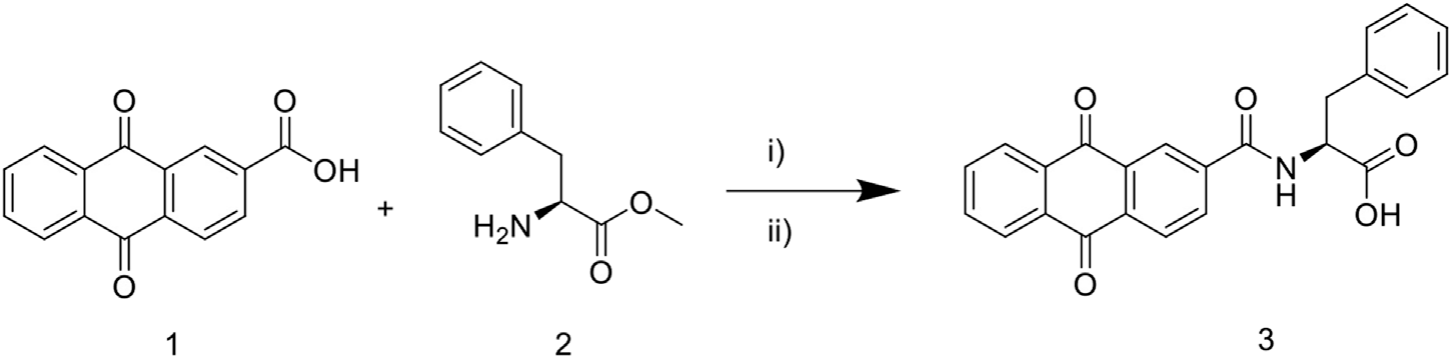
Synthetic route of 9, 10—anthraquinone XOD inhibitor. Scheme 1: reagents and conditions: i) EDCI, HOBt, CH_2_Cl_2_, 25°C, overnight; ii) MeOH/THF, NaOH, 50°C, 4 h; 10% HCl, pH 3.

The synthetic route to target compound 3 (NAY) is shown in Figure 1. Compounds 1 and 2 were easily synthesized, according to the literature reported methods (Ashnagar and Bruce, 2011; Zhang et al., 2018). A mixture of 1 (2.52 g, 10 mmol) and 2 (1. g, 10 mmol) and HOBt (0.135 g, 1 mmol) were dissolved in CH_2_Cl_2_ (40 ml) and stirred at 25°C for 15 min, then EDCI (2.87 g, 15 mmol) were added immediately. The reaction mixture was then stirred at 25°C overnight. After the reaction is complete, the mixture was concentrated under reduced pressure, then alcohol (10 ml) was added and poured into cold water (200 ml), filtered, and dried, and the solid was used directly without further purification (Sun et al., 2015).The solid was dissolved in a mixed solution (10ml, MeOH/THF = 1/1) and stirred at 25°C for 15 min, then 10 M NaOH (10 ml) was dropped, and the mixture was stirred at 50°C for 4 h. Then, the reaction mixture was diluted with water (50 ml) and adjusted pH to 3 with 10% HCl. The formed precipitate was collected and purified by recrystallization to provide pure compound 3 (Zhang et al., 2018).

## 2 Materials and animals

### 2.1 Animals and cells

SPF male and female KM mice (6–8 weeks old, 20 ± 2 g) were purchased from Jiangsu Huachuang Xinnuo Pharmaceutical Technology Co., Ltd. (Permit ID: SCXK(SU)2020-0009, certificate No.: 202103492). Animals were under a normal 12-h/12-h light/dark schedule with the lights on at 07:00 a.m. They were housed at room temperature (22 ± 2°C) with relative humidity (55 ± 5%) and given a standard chow and water *ad libitum*. A total of 12 SPF grade SD rats, male, were provided by the Experimental Animal Management Department of Shanghai Institute of Family Planning Science. (Permit ID: SCXK(HU)2018-0006, certificate No.: 20180006026509). Before administration, the rats would be adaptively fed for 1 week in the Experimental Animal Center Laboratory of China Pharmaceutical University. During the experiment, the rats had normal diet and water and being maintained with the circadian rhythm cycle. The experimental plan was approved by the Animal Protection and Ethics Committee of China Pharmaceutical University.

Alpha mouse liver 12 (AML12) and human renal proximal tubule (HK-2) cells were provided by Cell Bank of Chinese Academy of Sciences (Shanghai, China). The cells were cultured according to the reference culture conditions provided by Shanghai Cell Bank.

### 2.2 Chemicals and materials

Fetal bovine serum (FBS), phosphate balanced saline (PBS), BCA protein assay kit, 4% paraformaldehyde Solution, mouse IL-1β, IL-6, TNF-α, and NLRP3 ELISA Kits were from Nanjing Senbega Biotechnology Co., Ltd. (Nanjing, China). Penicillin–streptomycin were obtained from MCE. Adenosine and xanthine oxidase were purchased from Sigma-Aldrich Chemical Company (St. Louis, Missouri, United States). Potassium oxonate and allopurinol were purchased from Shanghai Aladdin Biochemical Technology Co., Ltd. (Shanghai, China). UA, BUN, CRE, and XOD determination kits were purchased from Nanjing Jiancheng Biotechnology Research Institute (Nanjing, China). RPMI-1640 and DMEM/F12 medium and PageRuler prestained protein ladder were from Thermo Fisher Technology Co., Ltd. (DMEM; Gibco, Grand Island, NY, United States). Anti-GLUT9, anti-OAT1, anti-OAT3, anti-caspase 1, anti-β-actin, anti-GAPDH, and HRP-conjugated affinipure goat antirabbit IgG were purchased from Proteintech Group, Inc. (Wuhan, China). Anti-XOD was purchased from Abcam (Cambridge, MA, United States), anti-NLRP3 and anti-ASC were purchased from Affinity (Chicago, IL, United States). Protease inhibitor cocktail and RIPA buffer were purchased from New Cell and Molecular Biotech Co., Ltd. (Suzhou, China). PVDF membrane was purchased from Bio-Rad Laboratories, Inc. (California, United States). Both SDS-PAGE running buffer (powder) and SDS-PAGE transfer buffer (powder) were bought from Servicebio Technology Co., Ltd. (Wuhan, China). PAGE Gel Fast Preparation Kits were purchased from Shanghai Epizyme Biomedical Technology Co., Ltd. (Shanghai, China). Primary antibody dilution buffer, secondary antibody dilution buffer, and blotting grade were purchased from Beyotime Biotechnology Co., Ltd. (Shanghai, China).

## 3 Methods

### 3.1 Toxicology experiment

#### 3.1.1 Acute toxicity evaluation

The method of Lorke (1983) was used for the evaluation of acute toxicity. The 28 male Kunming mice selected for the acute toxicity studies were divided into seven groups of four mice each. The animals were fasted overnight before commencement of NAY. The dosage of mice in seven groups were 0, 50, 100, 500, 1000, 2500, and 5,000 mg/kg, respectively. The animals were maintained under observation for 24 h for signs of toxicity and death (Lorke, 1983).

#### 3.1.2 Subacute toxicity study

A total of 30 male and 30 female mice were selected randomly and then divided into 12 groups of five mice each. Female and male rats were divided into six dose groups. The dosage of the six dose groups were 0, 50, 100, 500, 1000, and 2500 mg/kg, respectively. All drug administration was performed consecutively for 7 days after which the animals were sacrificed.

The blood was taken from the eyes of mice and sacrificed by neck amputation, then the heart, liver, spleen, lung, kidney, and brain tissues of mice were separated quickly on the ice platform, and the tissues were weighed. Tissue weight was used to calculate the organ coefficient. The dissected organs were fixed in paraformaldehyde for later pathological sections. One drop of mouse blood was taken into an anticoagulant tube for the detection of blood routine, and the remaining blood was centrifuged at 3000 rpm for 10 min to obtain serum for the detection of biochemical indexes such as uric acid.

Serum UA, CRE, BUN, and other biochemical levels and blood routine were determined by the hospital. Blood cells were counted using a hematology analyzer, and mice serum biochemical indexes were measured using an automated blood biochemistry analyzer.

The liver and kidney tissues fixed in paraformaldehyde were dehydrated to be transparent. Then put the transparent tissue block into the melted paraffin, and embed it after the paraffin is completely immersed in the tissue block. Fix the embedded wax block on the microtome and cut it into 5 μm thin slices. The thin slice is ironed in hot water, then pasted on the glass slide, and dried. Before staining, paraffin wax in the sections was removed with xylene, then soaked with high to low concentration alcohol, and finally soaked in distilled water for 2 min. The dewaxed and rehydrated slices were stained in hematoxylin solution for several minutes and then washed with running water. Then put the slices into 1% hydrochloric alcohol (5–10 s) and rinse with tap water, after rinsing, place the slices in distilled water. After 2 min, the slices were removed and dehydrated with high to low concentration alcohol. After dehydration, the slices were stained with alcohol eosin solution for 2–3 min. After dehydration by alcohol, xylene was used to make the slices transparent. Finally, the transparent slices were dripped with gum and sealed with a cover glass.

### 3.2 Pharmacological experiments

#### 3.2.1 Animal experiment

##### 3.2.1.1 Hyperuricemia mice and drug administration

Ninety SPF male KM mice (20 ± 2 g) were maintained in a normally controlled breeding room with standard laboratory food and water for 1 week prior to the experiments. The uricase inhibitor potassium oxonate was used to induce hyperuricemia in mice, according to previous reports (Chen et al., 2019).

Potassium oxonate (model drug), NAY (drug to be tested), and allopurinol (positive drug) were dissolved or suspended in double steaming water. The drug dose used is expressed in “mg/kg.” The dose given for each dose is converted based on the body surface area conversion method. Male Kunming mice were randomly divided into each group with eight mice, namely, blank control group, model control group (250 mg/kg potassium oxonate), extract model administration groups (low dose: 10 mg/kg, medium dose: 20 mg/kg, and high dose: 40 mg/kg), and positive control allopurinol model administration group (10 mg/kg). Except the normal control mice, others were orally administered by 250 mg/kg potassium oxonate once daily for seven consecutive days to induce hyperuricemia. From day 1 to day 7, it was administered daily at 9:00 a.m., mice were first given potassium oxonate, after 1 h, positive drug group was given allopurinol by gavage, experimental groups were given NAY by gavage, and normal control group was treated with distilled water. After 7 days’ treatment, diets were removed from the cages 12 h before the animals were sacrificed. The blood was allowed to clot for approximately 1 h at room temperature and centrifuged at 3,000 rpm for 10 min to obtain serum. Serum was divided into three portions, one to detect biochemical markers such as UA, one to detect inflammatory factors, and the remaining stored at−20°C as a backup. The livers and kidneys of mice were taken, some were immersed in paraformaldehyde, and the rest were stored at –80°C for subsequent detection.

##### 3.2.1.2 Biochemical analysis of hyperuricemia mice

The levels of serum UA, CRE, and BUN were measured by using the method recommended in the purchase kit. The levels of serum TNF-α, IL-6, and NLRP3 were measured by using the method recommended in the ELISA kits.

0.1 g mouse liver tissue was weighed accurately, and 0.9 g 0.9% normal saline was added. The mixture was mechanically homogenized under the condition of ice water bath to prepare 10% homogenate and centrifuged at 3000 rpm for 10 min to obtain supernatant for determination. The levels of XOD activities in liver were determined by the colorimetric method, using commercially available kits.

Accurately weigh 0.1 g of mouse kidney tissue, and then add 0.9 g PBS (PH7.4) to grind. Homogenize the specimen thoroughly with a homogenizer and centrifuge at 3000 rpm for 20 min for collecting the supernatant. The collected supernatant was divided into two parts, one to detect inflammatory factors and the other remained frozen at—20°C for further use. The levels of kidney TNF-α, IL-6, IL-1β, and NLRP3 were detected by using ELISA kits. The BCA kit was used to measure the concentration of renal protein and correct the expression level of renal inflammatory factors.

##### 3.2.1.3 Western blot analysis

The kidney tissue (100 mg) was mixed with radio immunoprecipitation assay (RIPA, 500 μL), containing phenylmethanesulfonyl fluoride (PMSF) and cocktail for homogenization. The homogenate was transferred into a 1.5 ml tube and placed on ice for 30 min. Then, centrifugation was carried out at 14,000 r/min for 10 min. The supernatant was collected into a 1.5 ml tube and stored at -80°C. A fraction of supernatant was mixed with loading buffer, followed by boiling for 10 min. The mixture was allowed to cool to room temperature and stored at -20°C after centrifugation. The protein concentration was determined with the BCA method. The protein sample was mixed with 1×loading buffer at a ratio of 1:1. Electrophoresis was performed at a constant voltage of 80 V for 120 min until the protein bands reaching the bottom of the gel. Then, the proteins were transferred onto the polyvinylidene fluoride membrane at a constant voltage for 1 h. The membrane was blocked in non-fat milk for 2 h at room temperature. Then wash the membrane in Tris-HCl Tween (TBST) thrice (5 min for each). The next step was treating the membrane with first antibody (ASC, 1:1000; Caspase-1, 1:2000; NLRP3, 1:2000; GLUT9, 1:2000; OAT1, 1:2000; OAT3, 1:2000, and β-actin, 1:5,000) at 4°C overnight. After washing in TBST thrice (5 min for each), the membrane was treated with HRP-conjugated secondary antibody (1:10000) at 37°C for 2 h. After washing in TBST thrice (5 min for each), chemiluminescent substrate was added onto the membrane followed by incubation in dark for 5 min, and the protein bands were detected by an ECL kit and analyzed by ImageJ software. The protein expression of ASC, Caspase-1, NLRP3, GLUT9, OAT1, and OAT3 in each group was normalized to that of β-actin.

Liver tissues were electrophoresed, transmembrane and other experiments were performed according to the same methods as appeal, the only difference was the use of XOD primary antibody to treat the membrane overnight at 4°C.

#### 3.2.2 Cell experiment

##### 3.2.2.1 Cell culture

AML12 cells were cultured according to the cell supplier’s instruction with slight modifications in DMEM medium supplemented with 10% fetal bovine serum (FBS), penicillin (100 IU/ml), and streptomycin (100 lg/ml) under atmosphere of 5% CO_2_/95% humidified air at 37°C.

HK-2 cells were cultured in RPMI 1640 medium with 10% FBS, 100 μg/ml streptomycin, and 100 units/mL penicillin. The cells were incubated at 37°C in a humidified atmosphere supplemented with 5% CO_2_. The culture medium was changed every other day.

##### 3.2.2.2 The safety range of NAY on AML12 cells and HK-2 cells

AML12 cells and HK-2 cells in the logarithmic growth phase were taken and placed in suspension. After being evenly blown, the cells were counted with a cell counter, and the concentration of cell fluid was diluted and adjusted to 10^5^ cells/ml. 100 µL of cell suspension was added to each well of the 96-well plate and then 100 µL of medium was added to each well. Then, the mixture was gently shaken and then cultured in a 5% CO_2_ cell incubator at 37°C for 24 h.

After 24 h, the cell culture medium was sucked out, washed with PBS for three times, and the incomplete medium with NAY was added. The final concentration of NAY was 1, 500, 250, 125, 67.5, and 0 μmol/L, which was put into 5% CO_2_ at 37°C cell culture box for 24 h. After 24 h, remove the media containing drugs, rinse with PBS for three times, and add CCK-8 detection solution. After adding CCK-8 detection solution, the 96-well plate was placed in a 5% CO_2_ cell culture box at 37 °C for 3 h. After 3 h of incubation, the enzyme plate was detected at 450 nm to calculate the cell survival rate.

##### 3.2.2.3 Induction of hyperuricemia

*In vitro* hyperuricemia model of AML12 cells and HK-2 cells was induced by adenosine and xanthine.

AML12 cells in logarithmic growth were digested with trypsin, and cell suspension was made by pipette blowing. After counting with a blood counting plate, the cells were diluted to 10^5^ cells/mL, and 1 ml of each well was inoculated in a medium dish. The cells were placed in a 5% (V/V) CO_2_ cell incubator at 37 °C for 24 h. 24 h later, the cell supernatant was discarded, washed with PBS for three times, and the adenosine solution prepared with the current basic medium without fetal bovine serum as the solvent was added. The dosage of adenosine was 1.5 mmol/L, and the cells were placed in the cell culture box for further incubation for 24 h. The culture dish was taken out, 0.005 IU xanthine oxidase (XO) was added to each well, and incubated in the cell incubator for 24 h, and then the hyperuricemia liver cell model was obtained.

According to the establishment method of HK-2 cells, hyperuricemia model was reported in the literature (Hou et al., 2019), and HK-2 cells was cultured in a complete medium in a 5% CO_2_, 37°C cell incubator. The cells in the logarithmic growth phase were inoculated on a 24-well plate at a concentration of 10^5^ cells/ml. The cells were cultured in a 37 °C incubator for 24 h. 24 h later, discard the culture medium, and wash cells by PBS for three times. Add 2.5 mmol/L adenosine solution prepared from the incomplete medium to each well and incubate in the incubator for 24 h. Then 0.005 IU/mg XO solution was added to each well in the 24-well plate, and the supernatant was collected after incubation for 12 h. The absorbance was measured on the microplate reader using the UA kit.

##### 3.2.2.4 Effect of NAY on decreasing UA level in AML12 cells and HK-2 cells

After cell safety range test, the final dose concentration range was determined to be 50–200 μmol/L.

AML12 cells were cultured in complete medium at 37 °C and a 5% CO_2_ cell incubator. Logarithmic growth cells were made into cell suspension with trypsin. The cells were inoculated into 24-well plates at the concentration of 10^5^ cells/ml and cultured in a cell incubator at 37°C and 5% CO_2_ for 24 h. After the cells adhered to the wall, the medium in 24-well plates was discarded and washed with PBS for three times. NAY solution (50, 100, and 200 μmol/L) and allopurinol solution (100 μmol/L) prepared with serum-free medium were added to the administration group, and serum-free medium was added to the blank control and model groups. After 24 h, the model and administration groups were washed with PBS for three times. The model and administration groups were added with 2.5 mmol/L adenosine solution prepared by serum-free medium and incubated in incubator for 24 h. Then 0.005 IU/mg XO solution was added to each well in 24-well plates. After incubation for 12 h, collect the supernatant to detect the level of UA and XOD activity of AML12 cells.

Hk-2 cells were cultured in a 5% CO_2_ cell incubator at 37 °C in complete medium. The cells in the logarithmic growth phase were inoculated on a 24-well plate at a concentration of 10^5^ cells/ml and cultured in a 5% CO_2_ cell incubator at 37°C for 24 h. After 24 h, the culture medium was discarded and washed with PBS for three times. The blank control and model groups were added with incomplete medium, and the drug administration groups were added with NAY (50, 100, and 200 μmol/L) and allopurinol solution (100 μmol/L) in an incubator for culture. After 24 h, the model and drug administration groups were washed with PBS for three times, and the model and drug administration groups were added with 2.5 mmol/L adenosine solution prepared with incomplete whole medium and incubated in an incubator for 24 h. Then add 0.005 IU/mg XO solution to each well in the 24-well plate. After 12 h of incubation, the supernatant was collected to detect the UA level.

##### 3.2.2.5 Effect of NAY on decreasing TNF-α, IL-6, IL-1β, and NLRP3 levels in HK-2 cells

The culture, induction, and administration of hyperuricemia HK-2 cells were the same as described earlier. Finally, the supernatant of the culture medium was collected to detect the levels of inflammatory factors. ELISA kits were used to detect the supernatant TNF-α, IL-6, IL-1β, and NLRP3 levels of the culture medium.

##### 3.2.2.6 Western blot analysis

The total protein of HK-2 cells and AML12 cells were extracted with total protein extraction kit, and the concentration was determined by the BCA kit. Electrophoresis, membrane transfer, antibody incubation, and other operations were performed in accordance with the Western blot experiment procedures. The protein bands were quantified by ImageJ software and normalized to individual β-actin expression levels.

### 3.3 Pharmacokinetics of NAY and allopurinol in rats

A total of 12 SD rats were randomly divided into four groups with three in each group; the four groups were intravenous NAY group (5 mg/kg), intravenous NAY group (50 mg/kg), intravenous allopurinol group (5 mg/kg), and allopurinol group (50 mg/kg). The volume of intravenous administration was 1 ml/250 g, and the volume of oral administration was 1 ml/100 g. Whole blood was collected at 2, 5, 10, 20, 30, 60, 120, 240, 360, 480, 720, and 1440 min after intravenous administration. The rats’ blood was transferred to the heparinized test tube. The whole blood was centrifuged at 8,000 rpm for 5 min, and the plasma was separated and stored in a refrigerator at -80°C for testing.

The HPLC-MS/MS method was established to determine the concentrations of NAY and allopurinol in rat plasma, respectively, and WinNonlin software was used to calculate the pharmacokinetic parameters.

### 3.4 Molecular docking of NAY

Molecular docking protein crystal XOD (PDB ID:1N5X) was downloaded from the PDB (https://www.rcsb.org/) database (Okamoto et al., 2003). Amino acid sequences of GLUT9 (UniProt ID: Q9NRM0), OAT1 (UniProt ID: Q4U2R8), and OAT3 (UniProt ID: Q8TCC7) were obtained from the UniProt database (https://www.uniprot.org/), and then modeled by AlphaFold Protein Structure Database (https://alphafold.ebi.ac.uk/) (Jumper et al., 2021; Varadi et al., 2022). The active site of XOD was determined using its original ligand febuxostat, and the active sites of Glut9, OAT1, and OAT3 were determined according to the methods described in the literature (Ferreira et al., 2019; Li et al., 2020). The software Autodock 4.0 was used to carry out molecular docking, according to the method reported in the literature (Steinberger et al., 2000), and then plotted using Pymol software (van Pouderoyen et al., 2001).

## 4 Results

### 4.1 Toxicological test results

#### 4.1.1 Acute toxicity

During the acute toxicity test, there were no deaths or any signs of toxicity observed after administration of NAY at 2,500 mg/kg, which was the no-observed-adverse-effect level (NOAEL). The dose of 5,000 mg/kg led to the death of one female and one male mouse within 24 h and on day 7, respectively. Therefore, the median lethal dose (LD_50_) of the extract was estimated to be more than 5,000 mg/kg. The body weight gain showed no significant difference between the control and treated groups. The macroscopic observation showed no remarkable pathological changes for the tested organs.

#### 4.1.2 Sub-acute toxicity

##### 4.1.2.1 General observations

No signs of death or toxicity were observed at doses below 500 mg/kg during 1 week repeated oral treatment. During intragastric administration, one male mouse died in the 1,000 mg/kg dose group and in the 2,000 mg/kg dose group, one female and one male died, respectively. Likewise, no statistically significant difference in body weight was found between the control and treated groups throughout the study period.

##### 4.1.2.2 Hematology analysis

Hematological parameters of the control and NAY-treated groups are presented in Table 1. Treatment of mice with the NAY for 1  week caused no significant changes in the hematological indices related to erythrocyte, leukocyte, and platelet. Across the blood routine data, only basophil and mean hemoglobin concentrations in the 2,000 mg/kg dose group and 1,000 mg/kg dose groups showed slight decrease.

**TABLE 1:** Effect of NAY on blood routine in mice.

Group	White blood cell (10^9^/L)	Monocyte (10^9^/L)	Basophils (10^9^/L)	Red blood cell (10^12^/L)	Hemoglobin (g/L)	Neutrophils (10^9^/L)
Control♂	4.70 ± 0.40	0.28 ± 0.09	0.46 ± 0.04	10.07 ± 0.19	149.75 ± 2.43	0.87 ± 0.16
50♂	4.92 ± 0.42	0.34 ± 0.06	0.55 ± 0.08	10.07 ± 0.22	145.75 ± 2.86	1.09 ± 0.21
100♂	5.27 ± 0.84	0.31 ± 0.12	0.52 ± 0.09	10.68 ± 0.59	157.00 ± 6.96	0.8 ± 0.12
500♂	5.42 ± 0.49	0.25 ± 0.06	0.37 ± 1.79	10.49 ± 0.21	155.00 ± 3.63	0.98 ± 0.11
1000♂	3.40 ± 0.29	0.14 ± 0.05	0.46 ± 0.04	9.78 ± 0.45	141.00 ± 5.80	0.74 ± 0.07
2000♂	4.58 ± 0.18	0.27 ± 0.06	0.44 ± 0.08	9.95 ± 0.30	145.75 ± 3.82	1.06 ± 0.12
Control♀	4.81 ± 0.40	0.27 ± 0.07	0.54 ± 0.06	10.68 ± 0.11	156.17 ± 2.68	0.63 ± 0.04
50♀	4.02 ± 0.29	0.13 ± 0.04	0.46 ± 0.06	9.97 ± 0.23	141.20 ± 2.67	0.72 ± 0.09
100♀	5.44 ± 0.48	0.22 ± 0.05	0.39 ± 0.03	10.31 ± 0.15	151.20 ± 2.46	0.72 ± 0.04
500♀	5.91 ± 0.75	0.16 ± 0.04	0.43 ± 0.07	10.59 ± 0.12	159.40 ± 2.18	0.74 ± 0.09
1000♀	3.96 ± 0.58	0.17 ± 0.05	0.57 ± 0.08	10.64 ± 0.32	157.40 ± 2.16	0.71 ± 0.13
2000♀	4.95 ± 0.33	0.27 ± 0.1	0.34 ± 0.04^#^	10.79 ± 0.52	156.75 ± 7.76	0.66 ± 0.06
Group	Mean corpuscular volume (fL)	Mean hemoglobin content (pg)	Mean hemoglobin concentration (g/L)	Platelet count (10^9^/L)	Mean platelet volume (fL)	Platelet specific volume (%)
Control♂	52.43 ± 0.98	14.85 ± 0.05	284.00 ± 5.20	1134.50 ± 106.20	7.25 ± 0.09	0.82 ± 0.07
50♂	51.08 ± 0.31	14.45 ± 0.03	283.50 ± 1.50	1019.00 ± 74.60	7.30 ± 0.17	0.74 ± 0.06
100♂	51.00 ± 0.79	14.62 ± 0.09	287.50 ± 2.65	976.60 ± 113.16	7.28 ± 0.17	0.69 ± 0.08
500♂	51.64 ± 0.48	14.74 ± 0.16	286.2 ± 2.27	1241.60 ± 45.36	7.16 ± 0.11	0.88 ± 0.03
1000♂	52.30 ± 1.10	14.43 ± 0.25	276.00 ± 3.49	994.25 ± 180.47	7.55 ± 0.06	0.75 ± 0.14
2000♂	50.98 ± 0.34	14.68 ± 0.13	287.50 ± 0.65	1238.25 ± 104.27	7.08 ± 0.09	0.88 ± 0.08
Control♀	49.8 ± 0.78	14.62 ± 0.19	293.67 ± 2.40	932.67 ± 51.27	7.12 ± 0.07	0.67 ± 0.04
50♀	50.48 ± 0.48	14.18 ± 0.19	280.80 ± 1.32	1123.40 ± 31.62	7.08 ± 0.14	0.80 ± 0.03
100♀	50.60 ± 0.14	14.68 ± 0.19	288.00 ± 3.29	906.00 ± 45.72	7.14 ± 0.12	0.65 ± 0.03
500♀	51.28 ± 0.48	15.06 ± 0.15	293.60 ± 0.75	980.40 ± 86.03	7.24 ± 0.11	0.71 ± 0.06
1000♀	50.46 ± 0.97	14.65 ± 0.24	268.60 ± 18.43^#^	860.00 ± 90.59	7.14 ± 0.07	0.61 ± 0.06
2000♀	49.98 ± 0.50	14.53 ± 0.07	290.50 ± 2.60	865.25 ± 69.07	7.03 ± 0.11	0.61 ± 0.04

Data were expressed as mean ± S.E.M.; *n* = 5.

Compared with control group, ^#^ *p* < 0.05, ^##^ *p* < 0.01, ^###^ *p* < 0.001.

##### 4.1.2.3 Biochemistry analysis

Table 2 summarizes the levels of biochemical factors in the control and treated mice. No significant differences among groups were found in the level of AST and ALT. Serum BUN levels were only slightly decreased in female mice treated with 2000 mg/kg NAY compared to the control mice (*p* < 0.05). Compared with the normal group, serum UA decreased significantly in all dose groups (both male and female). The CRE levels of male mice also decreased in all dose groups, but the CRE levels of female mice did not change significantly compared with the normal group. It is speculated that male mice are highly sensitive to NAY. CRE levels are lower in female mice than in male mice, so the reduction may not be significant.

**TABLE 2:** Effect of NAY on serum biochemical indices of mice.

Group	BUN (mmol/L)	UA (μmol/L)	CRE (μmol/L)	ALT (IU/L)	AST (IU/L)
Control♂	8.48 ± 0.72	193.67 ± 24.08	17.17 ± 1.62	46.17 ± 3.45	183.17 ± 33.12
50♂	8.07 ± 0.23	116.80 ± 19.81^##^	14.40 ± 0.68^#^	72.60 ± 13.64	205.40 ± 30.66
100♂	6.99 ± 0.48	108.40 ± 16.01^##^	13.80 ± 0.58^##^	48.40 ± 7.69	212.40 ± 24.04
500♂	8.70 ± 0.42	63.40 ± 6.91^###^	13.20 ± 1.11^##^	74.80 ± 12.77	199.40 ± 36.34
1000♂	7.00 ± 0.66	71.00 ± 11.60^###^	13.00 ± 0.71^##^	86.50 ± 12.85	159.25 ± 17.43
2000♂	7.87 ± 0.59	72.00 ± 16.56^###^	13.67 ± 0.33^#^	75.67 ± 4.26	226.33 ± 49.26
Control♀	8.13 ± 0.59	167.67 ± 23.15	13.83 ± 1.01	101.67 ± 39.13	200.67 ± 35.98
50♀	6.60 ± 0.68	125.80 ± 12.94	12.40 ± 0.60	49.60 ± 3.06	161.00 ± 18.34
100♀	7.48 ± 0.64	68.00 ± 8.26^###^	11.80 ± 0.20	60.60 ± 9.08	192.00 ± 29.53
500♀	9.16 ± 0.49	91.20 ± 17.83^##^	13.20 ± 0.86	66.60 ± 10.19	261.80 ± 42.65
1000♀	6.95 ± 0.46	74.50 ± 12.51^###^	12.50 ± 0.65	62.00 ± 5.94	185.00 ± 46.72
2000♀	6.04 ± 0.67^#^	39.25 ± 3.94^##^	12.75 ± 0.85	65.00 ± 7.11	229.25 ± 38.54

Data were expressed as mean ± S.E.M.; *n* = 5.

Compared with control group, ^#^ *p* < 0.05, ^##^ *p* < 0.01, ^###^ *p* < 0.001.

##### 4.1.2.4 The weight of body organs

The relevant data of coefficients of organs in mice after 1 week treatment with NAY are shown in Table 3. There were no significant differences in the weight of the liver, kidney, and brain of the NAY-treated mice as compared with the control animals at the end of the study. In the 2,000 mg/kg dose group, the organ coefficient of lung was significantly lower in both male and female mice than in the normal control group (*p* < 0.001). Except for the mice in the 50 mg/kg dose group, the heart coefficients of the female mice in the other dose groups were significantly lower than those in the normal group. The spleen coefficient of male mice in each dose group also decreased in varying degrees compared with the normal group.

**TABLE 3:** Effect of NAY on organ coefficient in mice.

Group	Heart (*10^−2^)	Liver (*10^−2^)	Spleen (*10^−2^)	Lung (*10^−2^)	Kidney (*10^−2^)	Brain (*10^−2^)
Control♂	0.543 ± 0.027	4.145 ± 0.225	0.427 ± 0.060	0.677 ± 0.077	1.086 ± 0.093	1.403 ± 0.043
50♂	0.524 ± 0.037	5.094 ± 0.328	0.307 ± 0.015^#^	0.746 ± 0.044	1.162 ± 0.023	1.546 ± 0.053
100♂	0.473 ± 0.021	4.336 ± 0.383^#^	0.308 ± 0.007^#^	0.751 ± 0.021	1.025 ± 0.028	1.519 ± 0.058
500♂	0.495 ± 0.026	4.584 ± 0.315	0.326 ± 0.015^#^	0.670 ± 0.034	1.123 ± 0.033	1.351 ± 0.052
1000♂	0.549 ± 0.039	5.005 ± 0.065	0.301 ± 0.016^#^	0.672 ± 0.037	1.100 ± 0.027	1.354 ± 0.055
2000♂	0.548 ± 0.028	4.335 ± 1.304	0.365 ± 0.043^#^	0.408 ± 0.085^###^	1.028 ± 0.034	1.468 ± 0.035
Control♀	0.614 ± 0.039	4.136 ± 0.202	0.339 ± 0.017	0.608 ± 0.068	0.981 ± 0.028	1.747 ± 0.048
50♀	0.535 ± 0.042	4.172 ± 0.213	0.377 ± 0.025	0.688 ± 0.037	1.043 ± 0.048	1.706 ± 0.113
100♀	0.470 ± 0.025^##^	4.693 ± 0.168	0.319 ± 0.025	0.713 ± 0.032	0.985 ± 0.019	1.572 ± 0.030
500♀	0.504 ± 0.023^#^	4.626 ± 0.176	0.306 ± 0.018	0.702 ± 0.031	1.019 ± 0.032	1.720 ± 0.101
1000♀	0.481 ± 0.027^#^	4.332 ± 0.164	0.332 ± 0.068	0.659 ± 0.010	0.974 ± 0.053	1.617 ± 0.048
2000♀	0.439 ± 0.064^##^	4.725 ± 0.247	0.304 ± 0.034	0.304 ± 0.034^###^	0.939 ± 0.090	1.665 ± 0.034

Data were expressed as mean ± S.E.M.; *n* = 5.

Compared with control group, ^#^ *p* < 0.05, ^##^ *p* < 0.01, ^###^ *p* < 0.001.

##### 4.1.2.5 Pathological observation of liver and kidney tissue in mice

**FIGURE 2:**
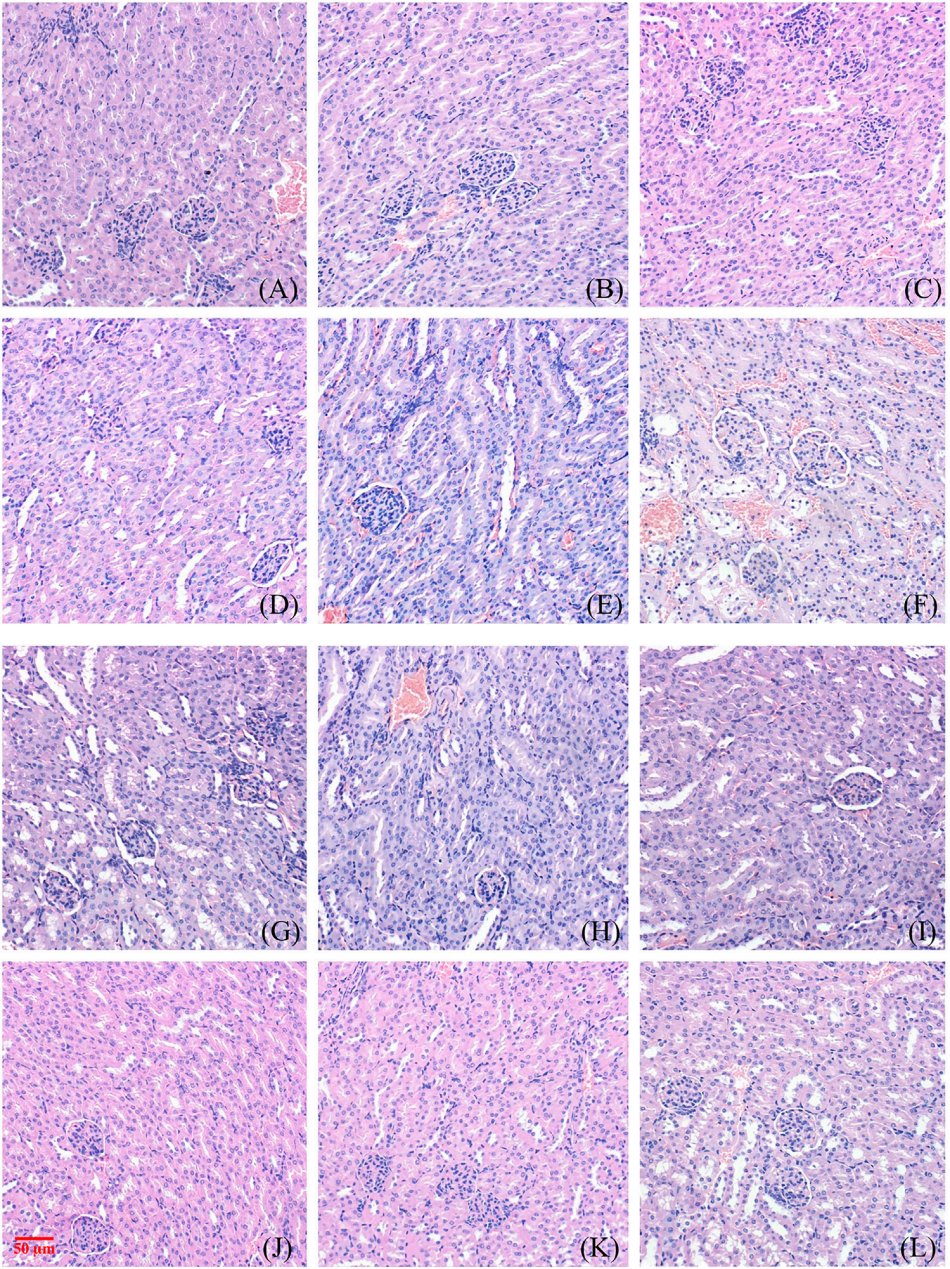
Toxic effect of different doses of NAY on renal tissue of mice. (**(A**–**F)** male mice and **(G**–**L)** female mice). [**(A)** 0 mg/kg; **(B)** NAY 50 mg/kg; **(C)** NAY 100 mg/kg; **(D)** NAY 500 mg/kg; **(E)** NAY 1,000 mg/kg; **(F)** NAY 2,000 mg/kg; **(G)** 0 mg/kg; **(H)** NAY 50 mg/kg; **(I)** NAY 100 mg/kg; **(J)** NAY 500 mg/kg; **(K)** NAY 1,000 mg/kg; and **(L)** NAY 2,000 mg/kg].

**FIGURE 3:**
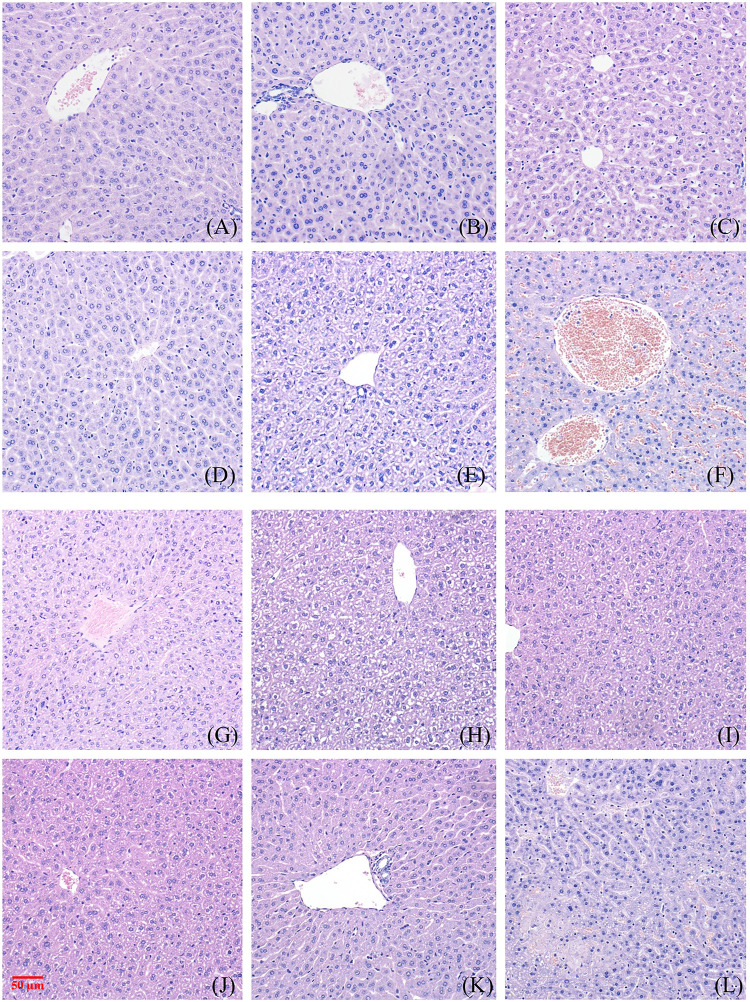
Toxic effect of different doses of NAY on liver tissue of mice. (**(A–F)** male mice and **(G–L)** female mice). [**(A)** 0 mg/kg; **(B)** NAY 50 mg/kg; **(C)** NAY 100 mg/kg; **(D)** NAY 500 mg/kg; **(E)** NAY 1,000 mg/kg; **(F)** NAY 2,000 mg/kg; **(G)** 0 mg/kg; **(H)** NAY 50 mg/kg; **(I)** NAY 100 mg/kg; **(J)** NAY 500 mg/kg; **(K)** NAY 1,000 mg/kg; and **(L)** NAY 2,000 mg/kg].

Figure 2 and Figure 3 show the effect of different concentrations of NAY on the renal and liver tissue structure of mice after long-term intragastric administration.

As shown in Figures 2A–D and Figures 2G–K, at this time the drug dose was less than 1000 mg/kg, the corticomedullary demarcation of the renal tissue is still clear, its intraglomerular and tubular distribution is still normal, and the glomeruli do not show definite sclerosis. No tubular luminal dilatation and clear tubular pattern were seen, and no significant inflammatory cell infiltration was seen in the interstitium. At the highest dose of 2,000 mg/kg, only mild edema of some renal tubular epithelial cells and unclear cytoplasmic structure of some renal tubular epithelial cells were found in Figure 2F and Figure 2L.

As shown in Figures 3A–D and Figures 3G–K, no obvious damage to the liver tissue was caused by NAY at doses of 1000 mg/kg and below. The arrangement of hepatocytes in the liver tissue was regular, the structure was clear, and there was no definite degeneration and necrosis in the tissue. Hepatic sinusoids showed no obvious dilatation. But in Figure 3F, in the 2000 mg/kg dose group, a small number of vacuoles were found in the cytoplasm of some hepatocytes of male mice, which tended to be steatosis. In Figure 3L, hepatocytes from female mice showed patchy necrotic foci and evidence of steatosis. Hepatic sinusoids showed no obvious dilatation. The interstitium was scattered with a few foci of inflammatory cells.

### 4.2 Pharmacological experiment results

#### 4.2.1 Animal experiment results

##### 4.2.1.1 Effect of NAY on XOD activity in liver of hyperuricemia mice

**FIGURE 4:**
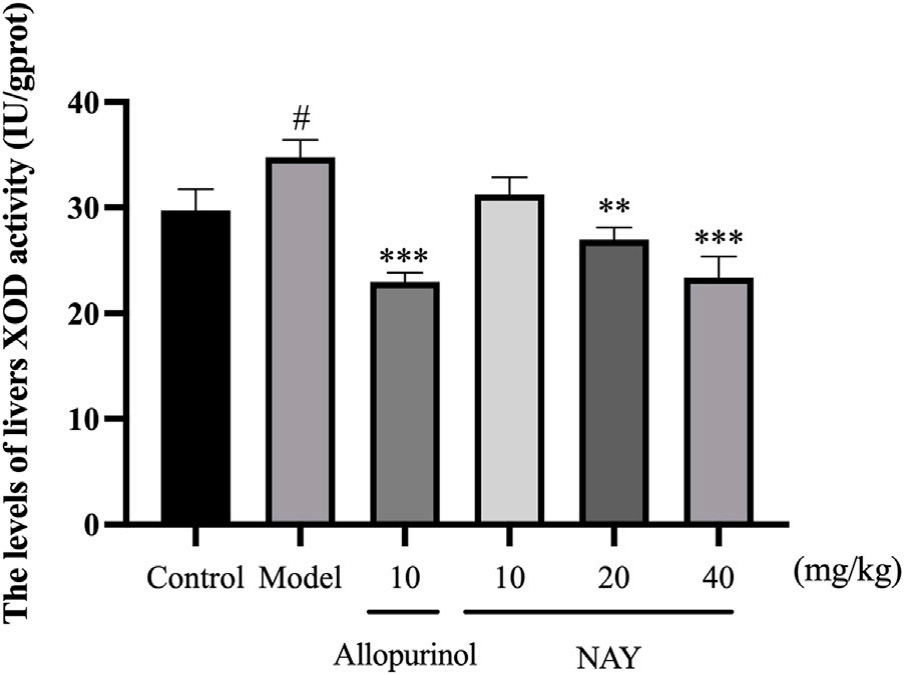
Effect of NAY on liver XOD activity in hyperuricemia mice. (Data were expressed as mean ± S.E.M.; *n* = 8. Compared with the control group, ^#^ *p* < 0.05, ^##^ *p* < 0.01, and ^###^ *p* < 0.001. Compared with the model group, ^*^ *p* < 0.05, ^**^ *p* < 0.01, and ^***^ *p* < 0.001).

The hyperuricemia model was successfully established by intragastric administration of potassium oxonate. As shown in Figure 4, compared with the control group, XOD activity in the model group significantly increased. Allopurinol, as a positive control drug, significantly decreased XOD activity compared with model group mice. Compared with the model group, both middle dose and high dose NAY can reduce XOD activity.

##### 4.2.1.2 Effects of NAY on UA, CRE, and BUN levels in serum and urine of hyperuricemia mice

**FIGURE 5:**
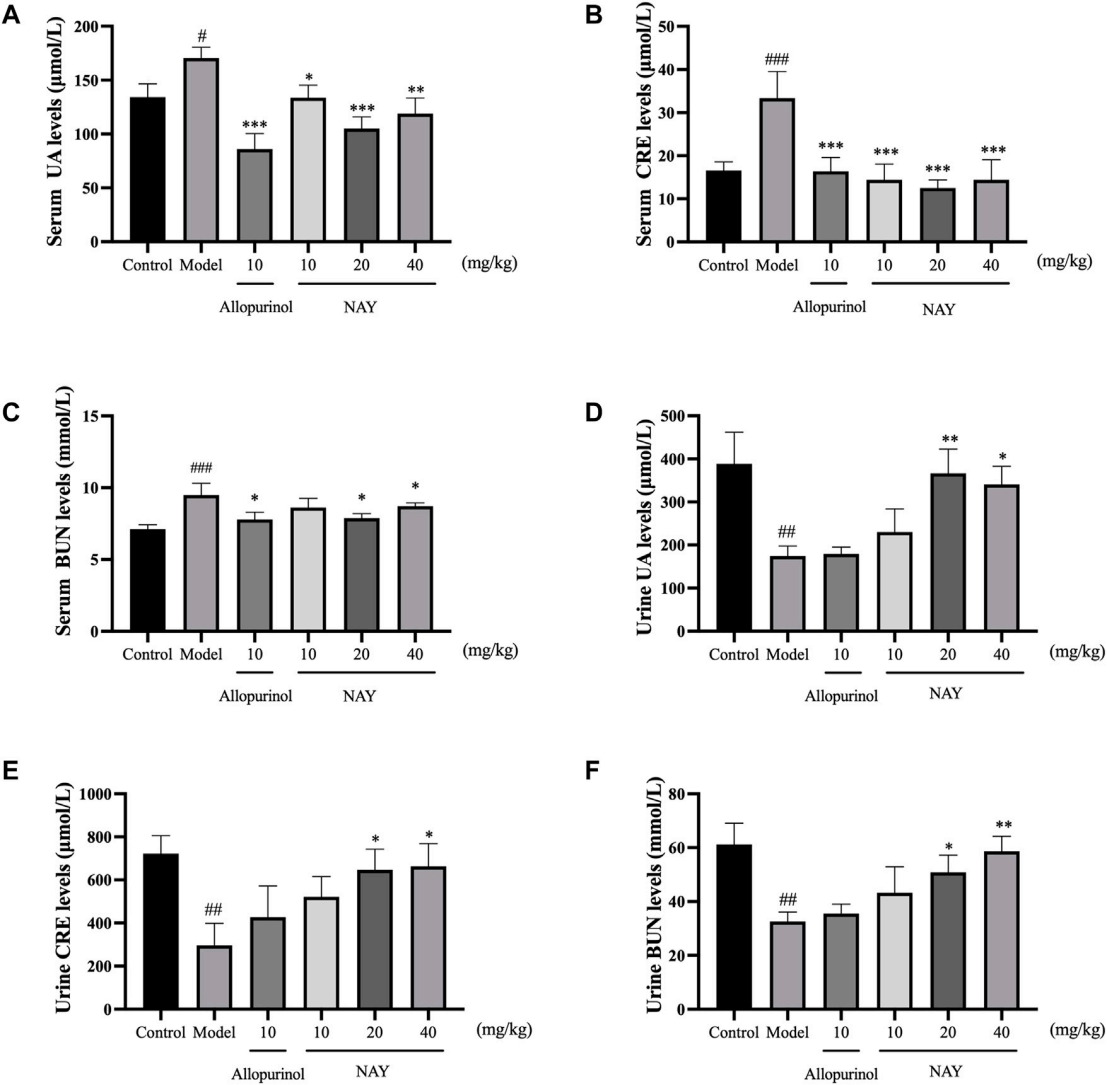
Effects of NAY on the levels of serum UA **(A)**, serum CRE **(B)**, serum BUN **(C)**, urine UA **(D)**, urine CRE **(E)**, and urine BUN **(F)** in hyperuricemia mice. (Data were expressed as mean ± S.E.M.; *n* = 8 (serum) or *n* = 5 (urine). Compared with the control group, ^#^ *p* < 0.05, ^##^ *p* < 0.01, and ^###^ *p* < 0.001. Compared with the model group, ^*^ *p* < 0.05, ^**^ *p* < 0.01, and ^***^ *p* < 0.001).

The hyperuricemia model was successfully established by intragastric administration of potassium oxonate. As shown in Figure 5, compared with the control group, the serum UA, CRE and BUN levels in the model group significantly increased. Allopurinol, as a positive control drug, obviously decreased the aforementioned indexes compared with model group mice. As shown in Figure 5A, compared with the model group, different doses of NAY can reduce the UA levels, and middle dose had the most significant reduction in UA levels (*p* < 0.001). In Figure 5B, compared with the model group, different doses of NAY can reduce the CRE levels, and the reduction was significant (*p* < 0.001).Finally, we can see from Figure 5C, compared with the model group, only the middle dose and high dose groups were able to reduce BUN levels slightly (*p* < 0.05).

As can be seen from Figures 5D–F, compared with the normal group, the excretion of UA, CRE, and BUN in hyperuricemia mice were significantly reduced (*p* < 0.01). The contents of UA, CRE, and BUN in the urine of mice were increased at each dose of the NAY administration group. The effect of the drug is dose-dependent, and high-dose NAY has the most significant effect on promoting excretion (UA and CRE: *p* < 0.05; BUN: *p* < 0.01).

##### 4.2.1.3 Effects of NAY on serum and kidney inflammatory factor levels of hyperuricemia mice

**FIGURE 6:**
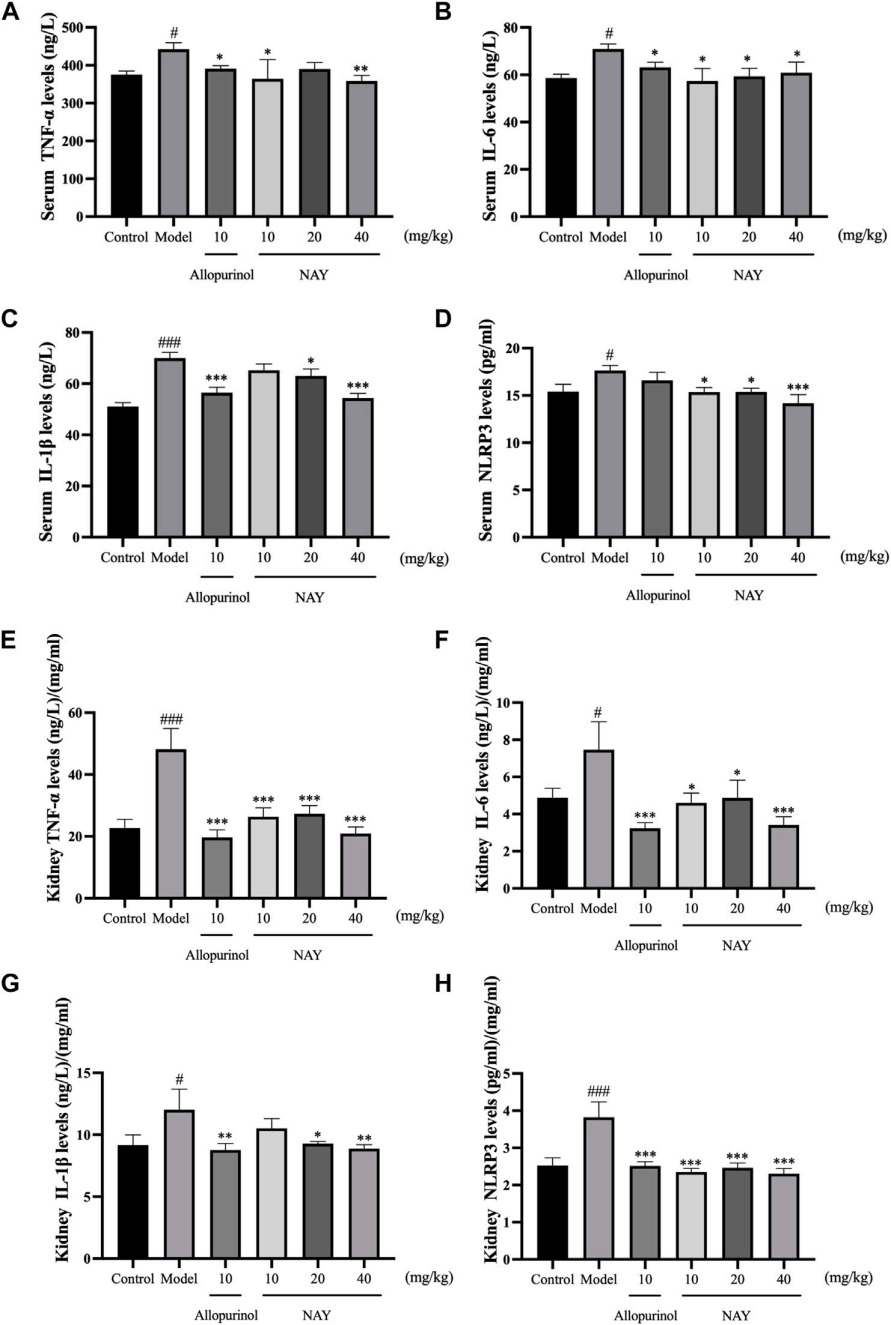
Effects of NAY on serum TNF-α **(A)**, IL-6 **(B)**, IL-1β **(C)**, and NLRP3 **(D)** levels in hyperuricemia mice. Effects of NAY on kidney TNF-α **(E)**, IL-6 **(F)**, IL-1β **(G)**, and NLRP3 **(H)** levels in hyperuricemia mice. (Data were expressed as mean ± S.E.M.; *n* = 8. Compared with the control group, ^#^ *P* < 0.05, ^##^ *P* < 0.01, and ^###^ *P* < 0.001. Compared with the model group, ^*^ *P* < 0.05, ^**^ *P* < 0.01, and ^***^ *P* < 0.001).

Hyperuricemia can cause inflammatory reaction in mice. As shown in Figure 6, compared with the normal control group, the model group not only increased serum TNF-α, IL-6, IL-1β, and NLRP3 levels but also increased the levels of TNF-α, IL-6, IL-1β, and NLRP3 in the kidney.

As shown in Figure 6A, compared with hyperuricemia mice, serum TNF-α levels were significantly reduced in the high dose group (*p* < 0.01). In Figure 6B, all dose groups of NAY can reduce the level of serum IL-6 in hyperuricemia mice, but the effect is not obvious (*p* < 0.05). In Figure 6C, high dose can significantly reduce the level of IL-1β in hyperuricemia mice (*p* < 0.001), and in Figure 6D, compared with the model group, the high dose group had a significant effect on reducing serum NLRP3 (*p* < 0.001).

As shown in Figure 6E, all dose groups of NAY can significantly reduce the level of renal TNF-α in hyperuricemia mice (*p* < 0.001). In Figures 6F,G, compared with other low dose groups, the high dose group had a better reduction of renal IL-6 and IL-1β levels, and in Figure 6H, compared with the model group, all dose groups of NAY can significantly reduce the level of renal NLRP3 levels (*p* < 0.001).

##### 4.2.1.4 NAY affect protein levels of XOD, ASC, Caspase-1, NLRP3, GLUT9, OAT1, and OAT3 in hyperuricemia mice

**FIGURE 7:**
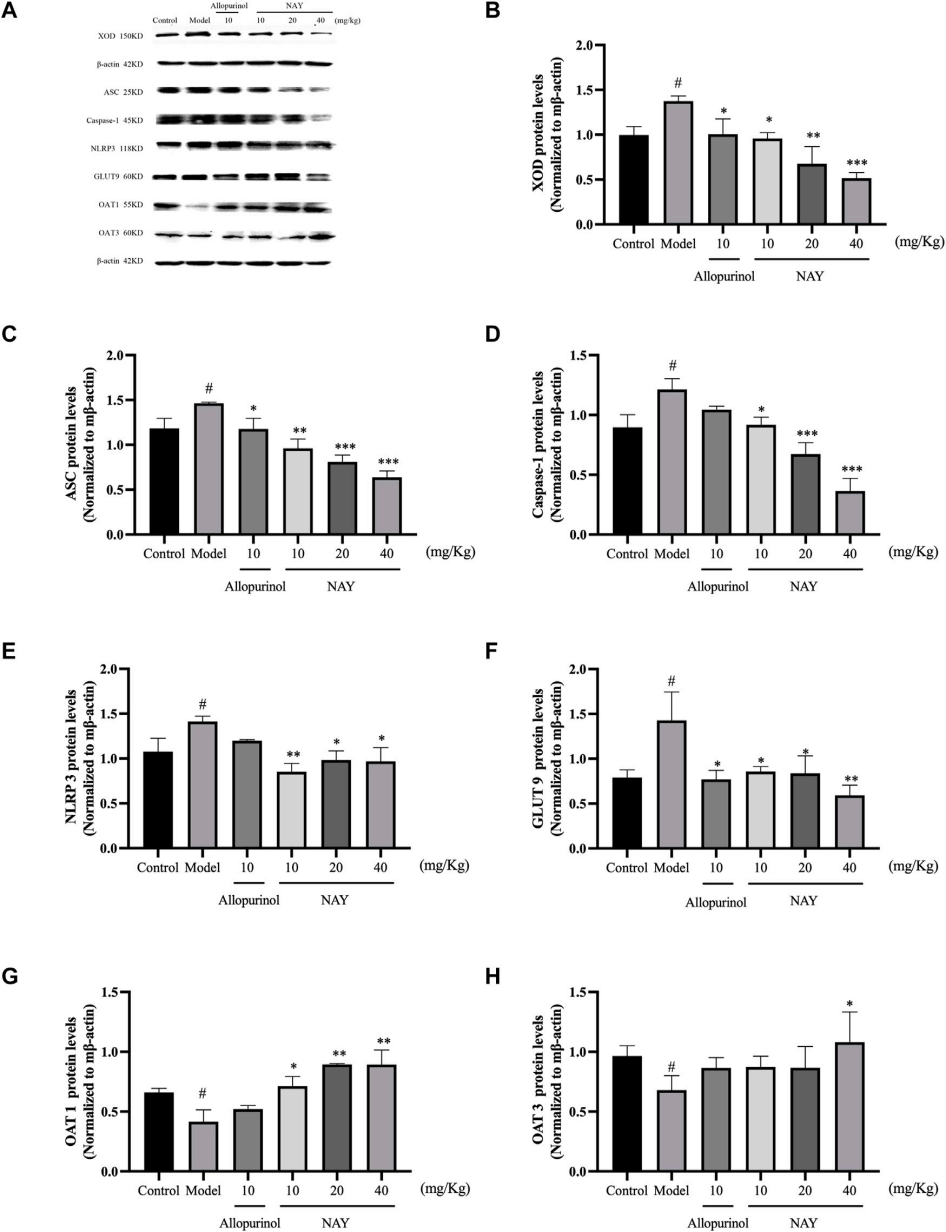
Effects of NAY and positive control on protein expression of XOD in liver tissues and protein expression of ASC, Caspase-1, NLRP3, GLUT9, OAT1, and OAT3 in renal tissues **(A)**. Data were normalized to β-actin **(B**–**H)**. (Data were expressed as mean ± S.E.M., *n* = 3. Compared with the control group, ^#^ *p* < 0.05, ^##^ *p* < 0.01, and ^###^ *p* < 0.001. Compared with the model group, ^*^ *p* < 0.05, ^**^ *p* < 0.01, and ^***^ *p* < 0.001).

The effects of NAY and allopurinol (positive control) on liver protein levels of XOD and renal protein levels of ASC, Caspase-1, NLRP3, GLUT9, OAT1, and OAT3 in hyperuricemia are shown in Figure 7. Potassium oxonate-induced hyperuricemia mice developed the elevated levels of liver XOD and renal ASC, Caspase-1, NLRP3, and GLUT9 protein and depressed OAT1 and OAT3 protein levels (all *p* < 0.05) compared with normal control mice.

NAY could downregulated the expression levels of liver XOD protein in hyperuricemia mice in a dose-dependent manner (*p* < 0.05, *p* < 0.01, and *p* < 0.001, respectively) compared with the model control group as shown in Figure 7B. As shown in Figures 7C–E, NAY successfully restored the overexpression of the renal NLRP3 inflammasome component in hyperuricemia mice induced by potassium oxonate (10 mg/kg: ASC and NLRP3, *p* < 0.01, Caspase-1, *p* < 0.05; 20 mg/kg: ASC and Caspase-1, *p* < 0.001, NLRP3, *p* < 0.05; 40 mg/kg: SC and Caspase-1, *p* < 0.001, NLRP3, *p* < 0.05). As shown in Figure 7F, the protein levels of GLUT9 could be downregulated, and the reduction was best at high doses (40 mg/kg, *p* < 0.01). As shown in Figure 7G, the protein levels of OAT1 could be upregulated with dose-dependence by NAY (*p* < 0.05, *p* < 0.01, and *p* < 0.01, respectively). But as shown in Figure 7H, NAY showed almost no effect on protein levels of OTA3, only the high dose group had a slight elevating effect (*p* < 0.05).

##### 4.2.1.5 Morphological manifestations of renal and liver tissue in mice

**FIGURE 8:**
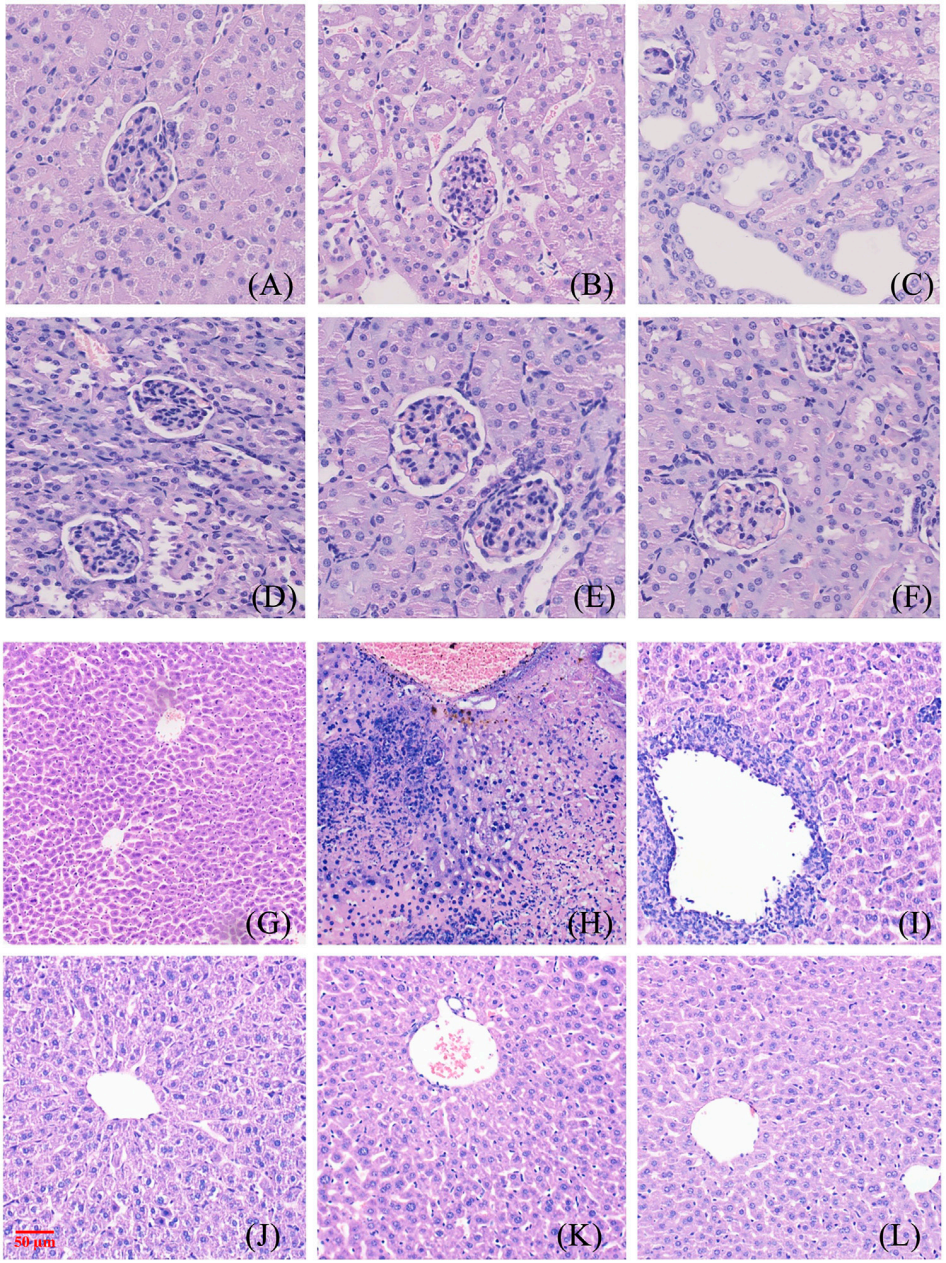
Morphological manifestations of renal and liver tissue in mice. [**(A)** renal control; **(B)** renal model; **(C)** renal allopurinol 10 mg/kg; **(D)** renal NAY 10 mg/kg; **(E)** renal NAY 20 mg/kg; **(F)** renal NAY 40 mg/kg; **(G)** liver control; **(H)**: liver model; **(I)** liver allopurinol 10 mg/kg; **(J)** liver NAY 10 mg/kg; **(K)** liver NAY 20 mg/kg; and **(L)** liver NAY 40 mg/kg].

The glomeruli of the mice in the blank control group were normal, and the epithelial cells of the tubules were full and arranged in an orderly fashion, showing no tubular dilation, clear lumen, and no inflammation and fibrosis (Figure 8A). No difference was observed in the number of glomeruli in the model mice, the tubular lumen was dilated, and the epithelial cells were vacuolated, atrophic, and necrotic, with infiltration of inflammatory cells and proteinaceous and cellular tubules found in the tubular lumen (Figure 8B). Renal cortical inflammatory cell infiltration and marked tubular luminal expansion were observed in allopurinol treated mice. In the low-dose NAY group, there were no abnormal glomerulus and slightly dilated renal tubule lumen. Renal staining of mice treated with middle and high doses of NAY showed no difference compared with the blank control (Figures 8C–F).

Mouse liver tissues as shown in Figures 8G–L, hepatocytes and cords of hepatocytes within the lobules were clear, and the cytoplasm of hepatocytes was full in the null control mice (Figure 8G). In the model group, hepatocytes and hepatocyte cords within the lobules were not clear, and some hepatocytes showed patchy necrosis with chronic inflammatory cell infiltration (Figure 8H). The arrangement of hepatocytes in the allopurinol group of mice was still regular, and in some areas, there were many inflammatory cell infiltrates with a small number of hepatocyte necrosis (Figure 8I). Compared with the model group, the liver tissue in the NAY administration group showed relatively intact liver corpuscle and liver lobule, the inflammatory infiltration was relatively reduced, and the high-dose group had the most obvious effect (Figures 8J–L).

#### 4.2.2 Cell experiment results

##### 4.2.2.1 Effects of NAY on XOD activity and UA level of AML12 cells

**FIGURE 9:**
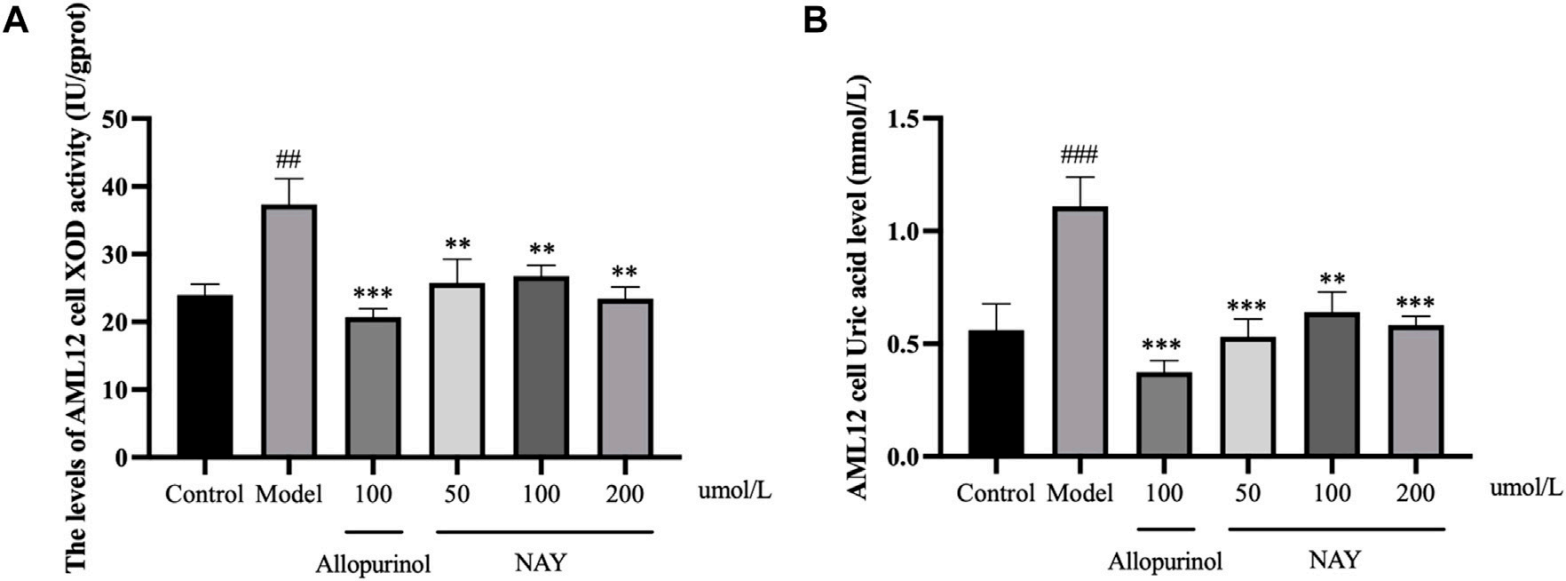
Effects of NAY on the levels of XOD activity **(A)** and culture medium UA **(B)** in AML12 cells. (Data were expressed as mean ± S.E.M.; *n* = 5. Compared with the control group, ^#^ *p* < 0.05, ^##^ *p* < 0.01, and ^###^ *p* < 0.001. Compared with the model group, ^*^ *p* < 0.05, ^**^ *p* < 0.01, and ^***^ *p* < 0.001).

As shown in Figure 9A and Figure 9B, compared with the normal group, the XOD activity of AML12 cells in the model group was enhanced evidently (*p* < 0.01) and the level of supernatant UA was significantly increased (*p* < 0.001), which proved that the hyperuricemia *in vitro* model was successfully constructed. Compared with the model group, both the effect of the allopurinol group on reducing XOD viability and the level of supernatant UA were obvious (*p* < 0.001). In Figure 9A, compared with the model group, XOD viability was significantly decreased in all treatment groups, which proved that the XOD activity was significantly inhibited (*p* < 0.01). In Figure 9B, all doses of NAY administration reduced the supernatant UA level, with a significant effect in the culture medium (50 μmol/L: *p* < 0.001; 100 μmol/L: *p* < 0.01; 200 μmol/L: *p* < 0.01).

##### 4.2.2.2 Effects of NAY on UA level in HK-2 cells culture medium supernatant

**FIGURE 10:**
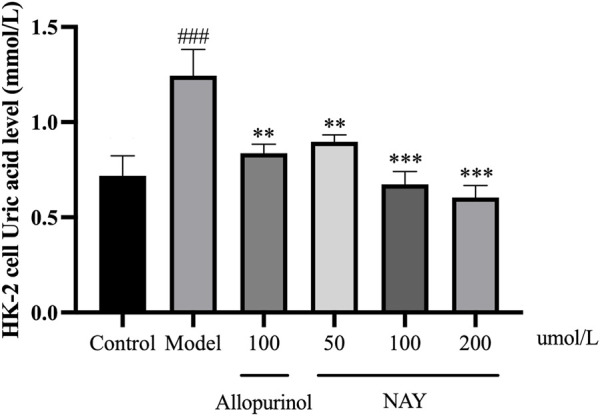
Effects of NAY on the levels of culture medium supernatant UA in HK-2 cells. (Data were expressed as mean ± S.E.M.; *n* = 5. Compared with the control group, ^#^ *P* < 0.05, ^##^ *P*<0.01, and ^###^ *P* < 0.001. Compared with the model group, ^*^ *P*<0.05, ^**^ *P*<0.01, and ^***^ *P* < 0.001).

The results in Figure 10 showed that compared with the normal group, the level of UA in the model group was significantly higher (*p* < 0.001), and the hyperuricemia cell model was successfully constructed. The level of UA in the positive drug group and NAY group was significantly decreased (50 μmol/L: *p* < 0.01; 100 μmol/L and 200 μmol/L: *p* < 0.01). Moreover, NAY decreased UA levels in a dose-dependent manner, and the highest reduction was observed in the high-dose group.

##### 4.2.2.3 Effect of NAY on TNF-α, IL-6, IL-1β, and NLRP3 levels in HK-2 cells culture medium supernatant

**FIGURE 11:**
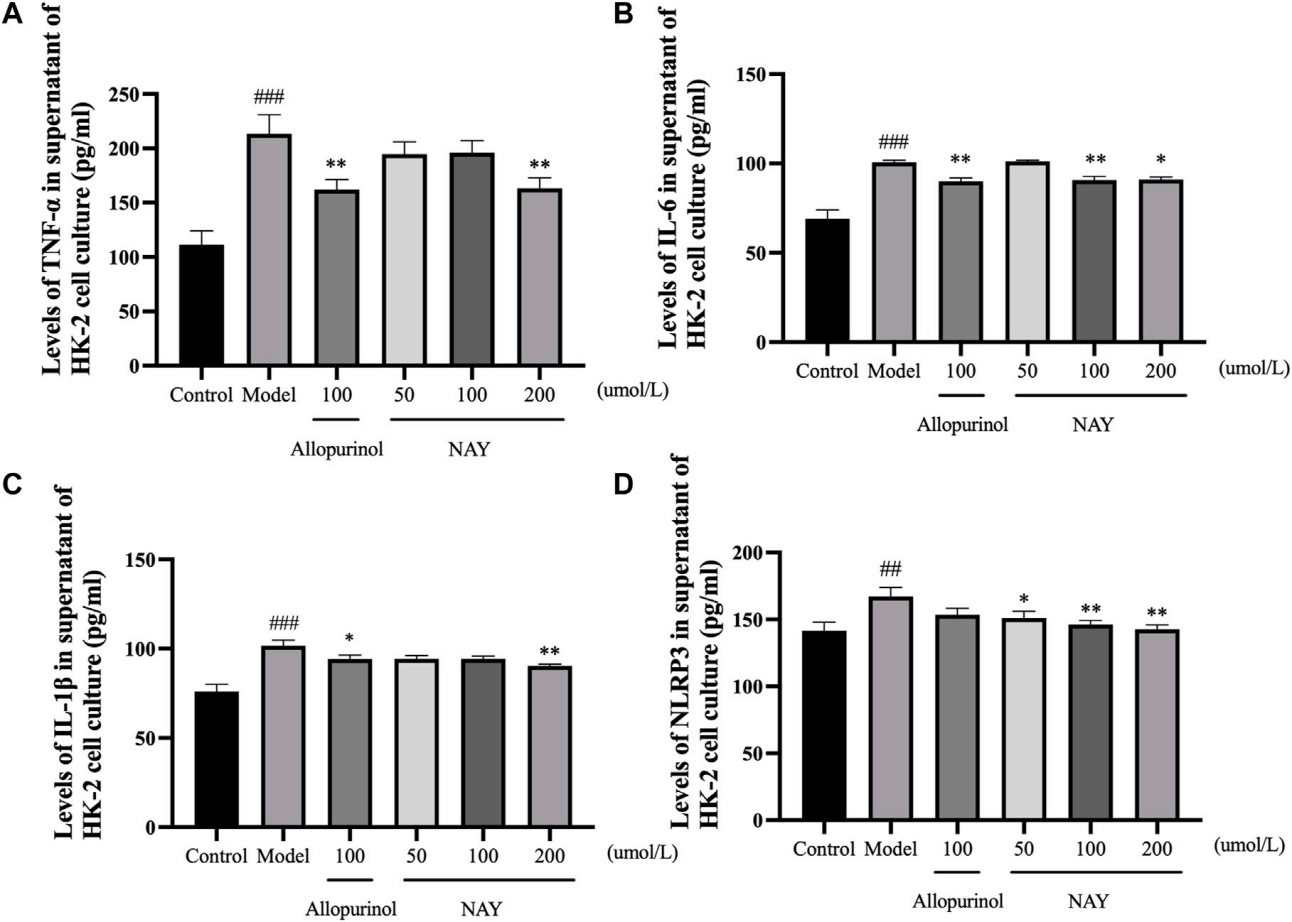
Effects of NAY on TNF-α **(A)**, IL-6 **(B)**, IL-1β **(C)**, and NLRP3 **(D)** levels in HK-2 cells culture medium supernatant. (Data were expressed as mean ± S.E.M.; *n* = 5. Compared with the control group, ^#^ *P* < 0.05, ^##^ *P* < 0.01, and ^###^ *P* < 0.001. Compared with the model group, ^*^ *P* < 0.05, ^**^ *P* < 0.01, and ^***^ *P* < 0.001).

The content of inflammatory factors in supernatant of HK-2 cells in each group was detected by ELISA as shown in Figure 11. The TNF-α, IL-6, IL-1β, and NLRP3 levels were significantly increased in the model group compared with the normal group (*p* < 0.01 or *p* < 0.001).

In Figure 11A, allopurinol and high-dose NAY significantly reduced the TNF-α level in medium supernatant (*p* < 0.01). In Figure 11B, allopurinol as a positive drug could reduce the IL-6 level (*p* < 0.01). The moderate and high-dose NAY groups also had a IL-6 level reduction (100 μmol/L: *p* < 0.01; 200 μmol/L: *p* < 0.05). In Figure 11C, compared with the model group, the level of IL-1β in culture medium supernatant of high dose group showed a downward trend (*p* < 0.01). In Figure 11D, all doses of NAY significantly reduced the levels of HK-2 NLRP3 (50 μmol/L: *p* < 0.05; 100 μmol/L and 200 μmol/L: *p* < 0.01).

##### 4.2.2.4 NAY affected the expression of XOD protein in AML12 cells and ASC, Caspase-1, NLRP3, GLUT9, OAT1, and OAT3 proteins in HK-2 cells

**FIGURE 12:**
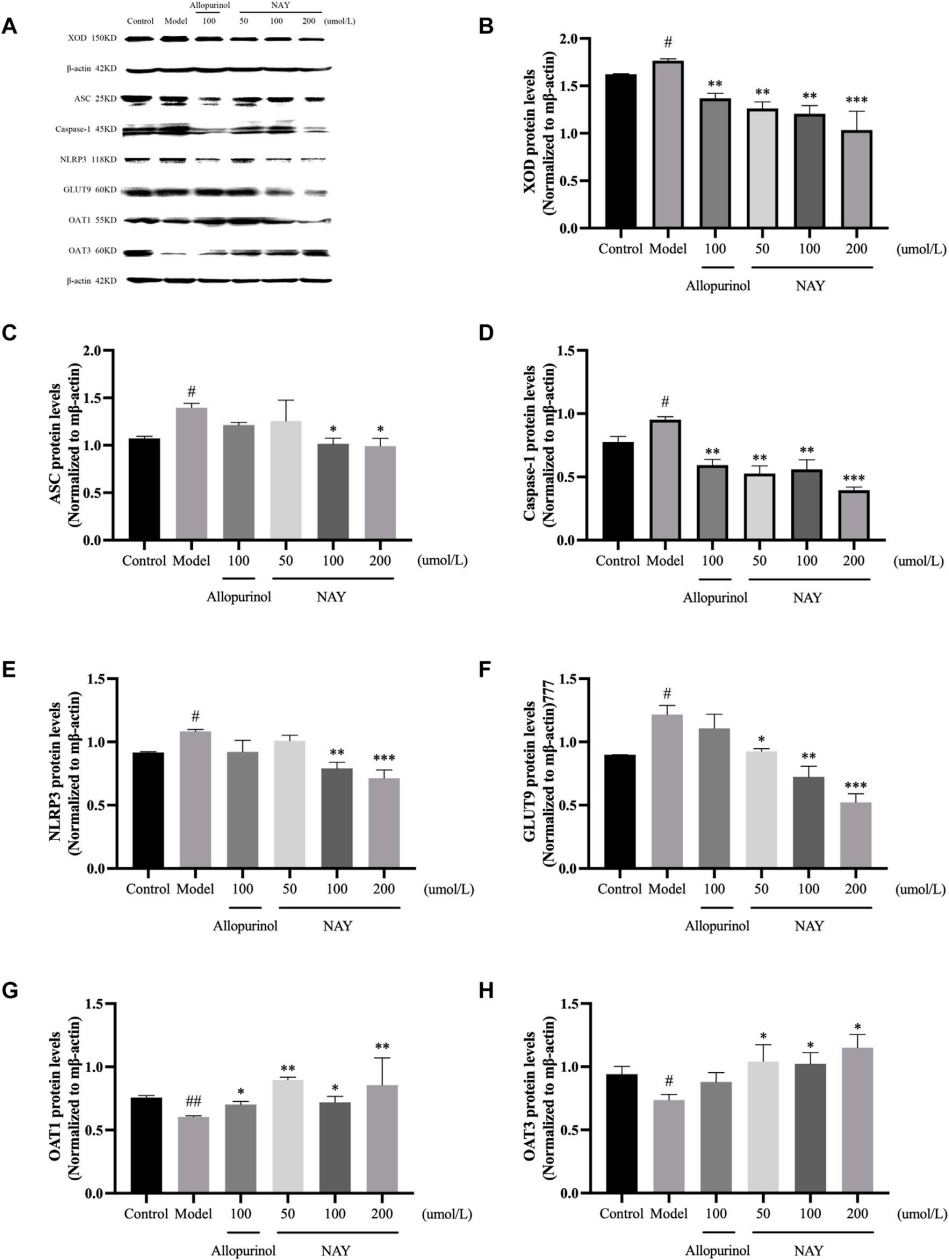
Effects of NAY and positive control on protein expression of XOD in AML12 cells and ASC, Caspase-1, NLRP3, GLUT9, OAT1, and OAT3 in HK-2 cells **(A)**. Data were normalized to β-actin **(B**–**H)**. (Data were expressed as mean ± S.E.M., *n* = 3. Compared with the control group, ^#^ *P* < 0.05, ^##^ *P*<0.01, and ^###^ *P* < 0.001. Compared with the model group, ^*^ *P* < 0.05, ^**^ *P* < 0.01, and ^***^ *P* < 0.001).

The effects of NAY and allopurinol on the expression of XOD protein in AML12 cells and ASC, Caspase-1, NLRP3, GLUT9, OAT1, and OAT3 protein in HK-2 cells are shown in Figure 12A.

As shown in Figure 12B, the expression level of XOD protein in the model group was significantly higher than that in the normal group (*p* < 0.05), and compared with the model group, the expression level of XOD protein in the allopurinol administration and NAY administration groups was significantly reduced (allopurinol: *p* < 0.01; 50 μmol/L and 100 μmol/L: *p* < 0.01; 200 μmol/L: *p* < 0.001).

In Figures 12C–E, the protein expression levels of ASC, Caspase-1, and NLRP3 in adenosine-induced hyperuricemia HK-2 cells were higher than those in the blank control group (*p* < 0.05), and NAY can alleviate the overexpression of ASC, Caspase-1, and NLRP3 proteins (50 μmol/L: Caspase-1, *p* < 0.01; 100 μmol/L: ASC, *p* < 0.05, NLRP3 and Caspase-1, *p* < 0.01; 200 μmol/L: ASC, *p* < 0.05, NLRP3 and Caspase-1, *p* < 0.001).

In Figure 12F, compared with the negative control group, the GLUT9 protein expression level of HK-2 cells in the model group increased (*p* < 0.05), and each dose of NAY could inhibit the increase of this protein expression level, and the inhibition degree was dose-dependent (50 μmol/L: *p* < 0.05; 100 μmol/L: *p* < 0.01; 200 μmol/L: *p* < 0.001).

In Figures 12G,H, OAT1 and OAT3 proteins, serving as uric-secreting proteins, showed a downward trend in protein expression induced by adenosine. However, t NAY could restore the abnormal expression of these two proteins (OAT1: 50 μmol/L and 200 μmol/L, *p* < 0.01; 100 μmol/L, *p* < 0.05; OAT3: *p* < 0.05).

### 4.3 Pharmacokinetics of NAY and allopurinol in rats

#### 4.3.1 Pharmacokinetics of NAY in rats

**FIGURE 13:**
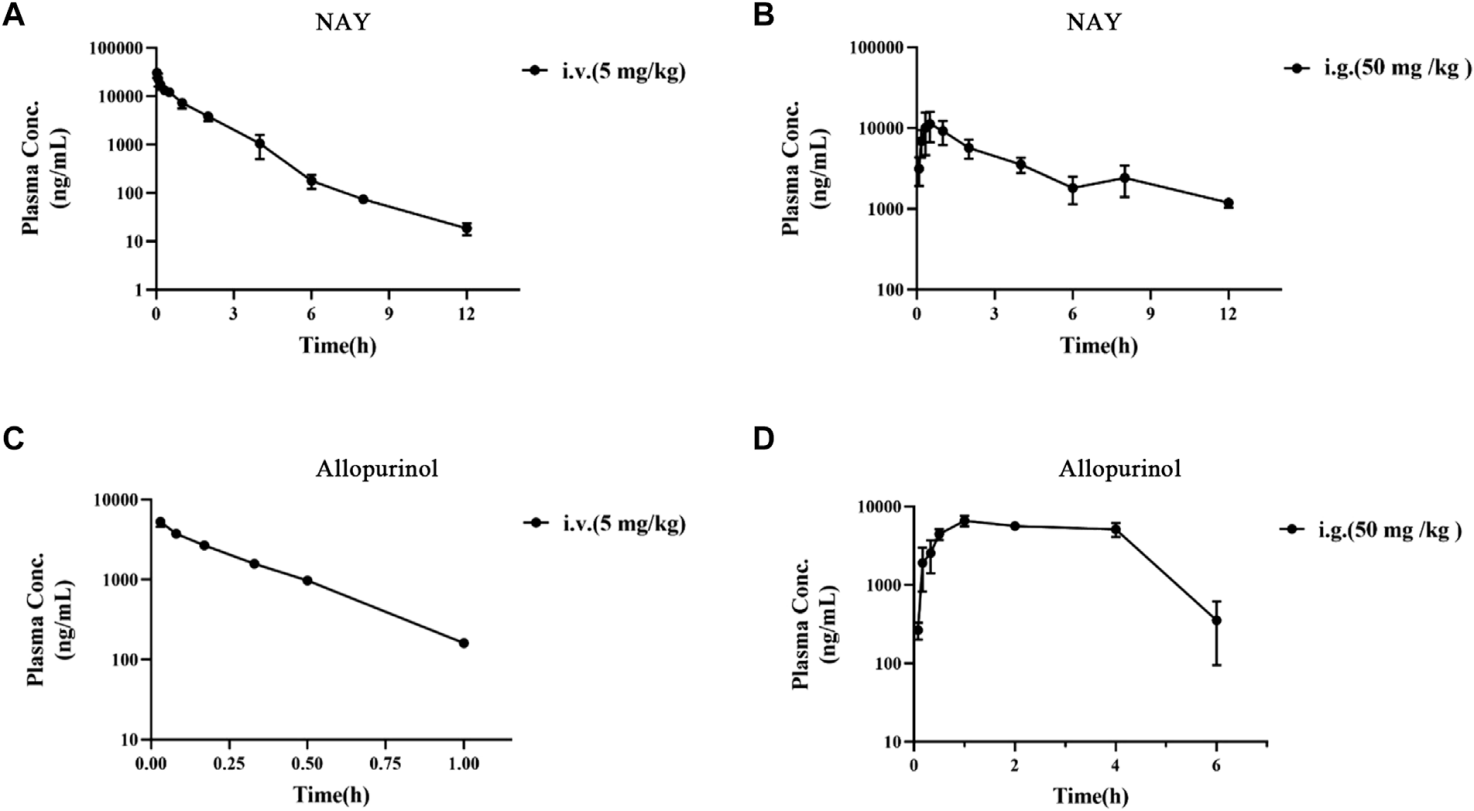
**(A)**: Blood concentration–time curve of NAY (5 mg/kg) intravenously injected in rats (*n* = 3). **(B)**: Blood concentration–time curve of NAY (50 mg/kg) after intragastric administration in rats (*n* = 3). **(C)**: Blood concentration–time curve of allopurinol (5 mg/kg) intravenously injected in rats (*n* = 3). **(D)**: Blood concentration–time curve of allopurinol (50 mg/kg) after intragastric administration in rats (*n* = 3).

The blood concentration time profiles of NAY after intravenous and intragastric administration in rats are shown in Figure 13A and Figure 13B. A randomized standard curve was prepared before measuring the amount of NAY in rat plasma samples taken at different times after administration of NAY, and the obtained regression equation was determined to be y = 0.0332739 x + 0.0660054, R^2^ = 0.9923, with a quantitative range of 5–5,000 ng/ml. Main pharmacokinetic parameters of NAY in rats are shown in Table 4. The drug concentration in plasma was higher after intravenous injection (C_2min_ = 30576.33 ± 6801.86 ng/ml), the drug was more slowly metabolized in blood, and the drug body exposure was greater (AUC_0-∞_ = 25812.42 ± 5,883.47 h μg/L). After intragastric administration, the concentration showed a second peak at 8 h. However, the second peak of plasma concentration did not occur in the intravenous injection group, so there may be no hepatoenteric circulation (hepatoenteric circulation generally occurs at 4–6 h), indicating that multiple sites of NAY may be absorbed in rats after intragastric administration.

**TABLE 4:** Main pharmacokinetic parameters of NAY after intravenous injection and gavage in rats (*n* = 3).

Parameter	Unit	1	2	3	Average	SD
(A): Intravenous injection						
AUC (0–t)	h·ug/L	32104.97	20434.61	24751.22	25763.60	5900.68
AUC (0–∞)	h·ug/L	32127.42	20485.62	24824.22	25812.42	5883.47
t1/2	H	1.09	2.10	2.07	1.76	0.58
V	L/kg	0.24	0.74	0.60	0.53	0.26
CL	L/h/kg	0.16	0.24	0.20	0.20	0.04
MRT (0–∞)	H	1.35	1.28	1.28	1.30	0.04
(B) Gavage						
AUC (0–t)	h·ug/L	25967.06	42942.19	51074.13	39994.46	12810.47
AUC (0–∞)	h·ug/L	39374.86	48183.41	58824.02	48794.10	9738.95
t1/2	h	4.07	3.37	4.13	3.86	0.42
Cmax	ug/L	6874.91	10854.05	16120.73	11283.23	4637.83
Tmax	h	0.50	0.50	0.50	0.50	0.00
V/F	L/kg	7.45	5.04	5.07	5.85	1.38
CL/F	L/h/kg	1.27	1.04	0.85	1.05	0.21
MRT (0–∞)	h	6.66	4.98	5.71	5.78	0.84
F (%)					18.90	

#### 4.3.2 Pharmacokinetics of allopurinol in rats

The blood concentration time profiles of allopurinol after intravenous and intragastric administration in rats are shown in Figures 13C,D. The accompanying standard curve was prepared before the allopurinol content in rat plasma samples was taken at different times after allopurinol administration. The regression equation was y = 0.00578645 x-0.00931793, R^2^ = 0.9930, and the quantitative range was 5–10,000 ng/ml. Main pharmacokinetic parameters of NAY in rats are shown in Table 5. After intravenous injection, the drug concentration in plasma was low (C_2_min = 5280.84 ± 686.48 ng/ml), the drug metabolism in blood was fast, and the drug exposure *in vivo* was small (AUC_0-∞_ = 1590.90 ± 138.60 h μg/L). The drug had a short half-life and a large elimination rate constant (about 0.20 ± 0.01 h, K = 3.47 h-1).

**TABLE 5:** Main pharmacokinetic parameters of allopurinol after intravenous injection and gavage in rats (*n* = 3).

Parameter	Unit	1	2	3	Average	SD
(A): Intravenous injection						
AUC (0–t)	h·ug/L	1599.82	1383.23	1648.60	1543.89	141.25
AUC (0–∞)	h·ug/L	1653.20	1432.09	1687.42	1590.90	138.60
t1/2	H	0.21	0.21	0.19	0.20	0.01
V	L/kg	0.91	1.05	0.81	0.92	0.12
CL	L/h/kg	3.02	3.49	2.96	3.16	0.29
MRT (0–∞)	h	0.27	0.29	0.27	0.27	0.01
(B) Gavage						
AUC (0–t)	h·ug/L	27475.25	19623.63	26462.91	24520.60	4271.00
AUC (0–∞)	h·ug/L	28658.23	20029.34	26666.72	25118.10	4518.09
t1/2	h	1.27	1.06	0.93	1.09	0.17
Cmax	ug/L	6104.81	5,968.97	7742.83	6605.54	987.26
Tmax	h	1.00	1.00	1.00	1.00	0.00
V/F	L/kg	3.19	3.81	2.53	3.18	0.64
CL/F	L/h/kg	1.74	2.50	1.87	2.04	0.40
MRT (0–∞)	h	2.88	1.84	2.35	2.36	0.52
F (%)					157.89	

### 4.4 Molecular docking results

**FIGURE 14:**
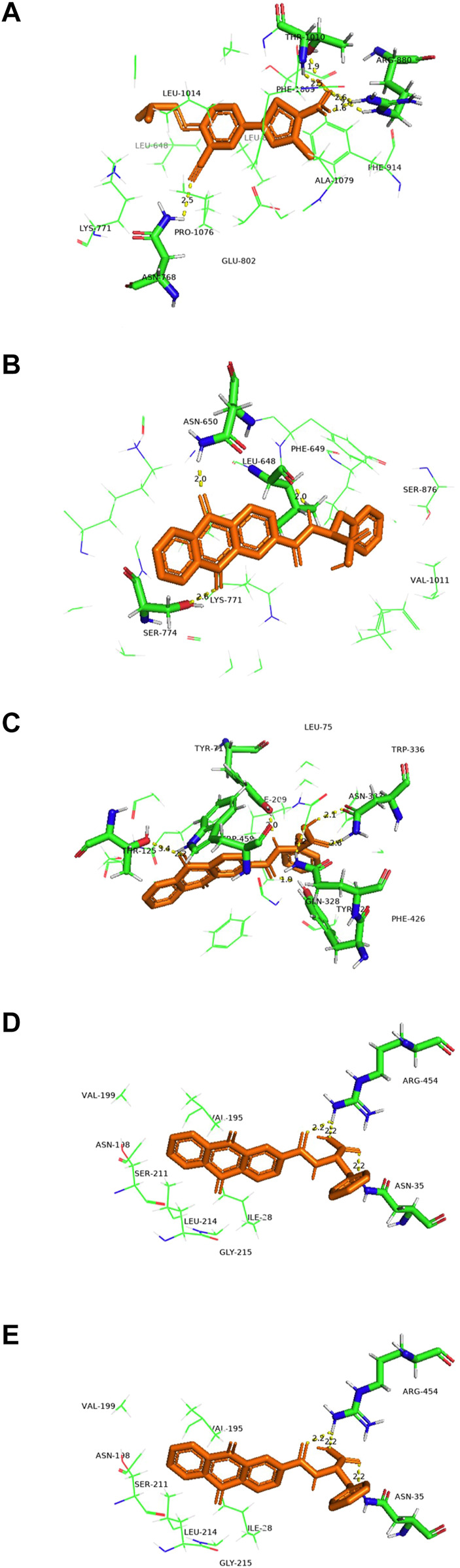
Active binding sites of NAY with receptor protein. [**(A)** Binding mode of XOD and febuxostatl. **(B)** Binding mode of XOD and NAY. **(C)** Binding mode of GLUT9 and NAY. **(D)** Binding mode of OAT1 and NAY. **(E)** Binding mode of OAT3 and NAY].

We docked febuxostat, the original ligand of XOD, to ensure the accuracy of the docking method. We docked febuxostat, the original ligand of XOD, to ensure the accuracy of the docking method. As shown in Figure 14A, the binding energy of fexostat to protein was -8.94 kcal/mol, and the cyano group in the fexostat structure and the carboxyl group on thiazole ring formed hydrogen bonds with amino acid residues Asn768, Thr1010, and Arg880 respectively, with bond lengths of 2.5, 1.6, 1.9, 2.4, and 2.6 Å. The docking sites were consistent with literature reports (Okamoto et al., 2003), demonstrating the accuracy of the present docking method. In Figure 14B, the binding energy of NAY to XOD was -6.25 kcal/mol, the carbonyl group on the anthraquinone ring and the amino group on amide bond formed hydrogen bonds with amino acid residues Asn-650, Ser-774, and Leu-648, respectively, with bond lengths of 2.0, 2.0, and 2.0 Å, respectively, and the docking sites were basically consistent with the report (Zhang et al., 2018). Through Figure 14C, the binding energy of NAY to GLUT9 is -9.85 kcal/mol. The carbonyl, amide, and carboxyl groups on anthraquinone formed hydrogen bonds with amino acid residues Thr-125, Trp-459, Tyr-71, Tyr-327, Asn-333, and Gln-328, respectively, with bond lengths of 3.4, 2.2, 2.0, 2.1, 2.6, and 1.9 Å. The docking sites were essentially the same as those reported in the literature (Ferreira et al., 2019), indicating that the compound NAY could work well with GLUT9, which was consistent with the previous pharmacological activities. Figure 14D shows that the binding energy of NAY and OAT1 is -8.48 kcal/mol. Benzyl was embedded into the side pockets formed by amino acid residues Ser-203, Met-31, Ala-200, Val-145, Leu-199, Gly-196, Asn-35, and Try-141. The carbonyl and carboxyl groups on anthraquinone formed hydrogen with amino acid residues Gly-223, Tyr-354, and Arg-466, and bond lengths were 2.8, 2.3, and 1.7 Å, respectively. The docking sites were basically consistent with those reported in the literature (Li et al., 2020), indicating that NAY could play a good role with OAT1, which was consistent with the previous pharmacological activity. Figure 14E shows that the binding energy of NAY and OAT3 was -8.64 kcal/mol. Anthraquinone skeleton was embedded into the pockets formed by amino acid residues Val-195, Val-199, Asn-198, Ser-211, Leu-214, Gly-215 and Ile-28. The amide bonds and carboxyl groups in the NAY structure formed hydrogen bonds with amino acid residues Arg-454 and Asn-35, respectively with bond lengths of 2.2, 2.2, and 2.2 Å. The docking sites were basically consistent with those reported in the literature (Li et al., 2020), indicating that NAY could play a good role with OAT3, which was consistent with the previous pharmacological activity.

## 5 Discussion

UA is the final product of catabolism of nucleic acid base purine, which can be divided into endogenous and exogenous. Endogenous purines are metabolized into UA *in vivo* by hypoxanthine-guanine phosphoribose transferase, xanthine invertase, and phosphoribose pyrophosphoamide conversion enzyme. Exogenous is the metabolism of purines in food into UA in the body. UA is excreted mainly by the kidneys. If the body produces too much UA or excretes too little UA from the kidney (urine), it will lead to the ultra-saturated state of urate in the extracellular fluid, which is a very insidiously harmful metabolic disease of human health. The serum UA level is not only directly related to gout, recent studies have found that hyperuricemia is often associated with traditional metabolic cardiovascular risk factors, such as hypertension, hyperlipidemia, diabetes, obesity, and insulin resistance and is an independent risk factor for cardiovascular disease (Hu et al., 2010; Kuo et al., 2010). At present, a number of antigout drugs, including anti-inflammatory drugs (indomethacin and colchicine) and the urate-lowering drugs (allopurinol and benzbromarone) are used as the primary clinical therapies to treat gout and hyperuricemia. Allopurinol is the most widely used first-line drug to control hyperuricemia via inhibiting XOD activity, and thus inhibiting the generation of UA from hypoxanthine (Schumacher et al., 2008). Indomethacin as the most potent non-steroidal anti-inflammatory drugs (NSAIDs) has been widely used in the treatment of acute gout to date (Shrestha et al., 1995; Rubin et al., 2004). However, few drugs produce satisfactory therapeutic efficacy without causing adverse effects, including gastric damage, renal toxicity, and hypersensitivity (Huh et al., 2013; Ryu et al., 2013; Arroyo-Lira et al., 2014; Yang et al., 2015), thus limit their clinical uses.

The most commonly used agent to reduce UA synthesis is allopurinol, which is a purine analog. Allopurinol not only affects the activity of other enzymes involved in purine and pyrimidine metabolism but also produces nephrotoxicity (Horiuchi et al., 2000). The use of allopurinol has certain limitations. First, the use of conventional doses of allopurinol cannot reduce serum UA to the normal level. Some gout patients, especially those with renal insufficiency, need to reduce the use of allopurinol, which is more difficult to reduce the serum UA to the target value. Moreover, allopurinol may also cause life-threatening allergic drug eruption (Hande et al., 1984; Singer and Wallace, 1986; Arellano and Sacristán, 1993). Through the pathological section, we can see that allopurinol has a certain injury effect on both the liver and kidney structures, which is speculated to have a large toxic effect. Although allopurinol urate lowering effect is significant, it is limited in its use due to toxicity. However, NAY showed better effects on restoring the structure of the liver and kidney in hyperuricemia mice, and the toxicity was significantly reduced compared with that of allopurinol. Pharmacokinetic results showed that NAY did not metabolize rapidly or distribute in tissues after entering the blood and did not metabolize weakly in rats. The half-life is significantly longer than allopurinol. It is noteworthy that after allopurinol gavage administration, the calculated bioavailability was 157.89%, which is much greater than 100%. Possibly because of the low plasma protein binding of allopurinol, plasma protein binding is saturated immediately after intravenous administration, and more of the free drug can be eliminated by metabolism or renal or other excretion. Whereas gavage is less prone to plasma protein saturability due to the presence of absorption processes that delay and reduce the rate and extent of drug entry into the bloodstream. As a result, allopurinol has low exposure and high bioavailability *in vivo* after intravenous administration. In conclusion, NAY have better therapeutic prospects than allopurinol. The wide safety range of NAY is conducive to its further development as a drug for hyperuricemia.

Anthraquinone (AQ), also known as 9,10-anthraquinone, refers to AQ compounds with the carbonyl group at C_9_ and C_10_, which are polycyclic aromatic hydrocarbon derivatives. In nature, AQ compounds are often found in higher plants such as Liao family, Rhamnaceae, Rubiaceae, Liliaceae, and Chinese medicinal materials such as rhubarb, aloe vera, and *Tripterygium wilfordii*. AQ has antitumor, diarrhea, antibacterial, antioxidant, diuretic, hemostatic, and other important pharmacological effects, especially in the prevention and treatment of cardiovascular diseases, senile dementia, cancer, AIDS, and other major diseases, which is widely used. This study proved for the first time that NAY has the effects of reducing UA and protecting the kidney in mice with hyperuricemia.

AQ has low acute toxicity. Studies have shown that AQ have an acute oral toxicity LD_50_ > 5,000 mg/kg BW in rats and mice. (Izmerov et al., 1982). Experimental results show that NAY has high safety. Acute toxicity tests did not cause death in mice, and in subacute toxicity tests, only isolated but not more than half of the mice died in 1000 and 2000 mg/kg dose groups. Long-term toxicity test showed that the highest dose of 2000 mg/kg had little effect on blood routine indexes of mice. The levels of glutamic-oxalacetic transaminase and glutamic-pyruvic transaminase in the administration group were not significantly different from those in the blank group, which further confirmed the wide safety range of NAY. It was observed by the pathological section that only the highest dose group (2000 mg/kg) caused certain damage to the liver and kidney tissue of mice, while the 1000 mg/kg dose group and below showed no obvious signs of damage to the liver and kidney tissue structure. In toxicological experiments, NAY was observed to reduce the serum CRE and UA levels. Then, combined with HE staining sections, it was speculated that NAY had a certain protective effect on the kidney in addition to its low toxicity.

UA elevation in the human body is mainly caused by purine metabolism disorder or UA excretion disorder. 70% of UA in the body is excreted by the kidneys with urine (Kou et al., 2016). In this animal experimental study, the level of serum UA in the hyperuricemia model group was significantly increased, indicating that the model was successfully constructed. In addition, it is believed that PO could seriously impair renal function. In this experiment, the BUN and CRE levels increased in the serum of the model group. After a week of treatment with different doses of NAY, compared with model

group, allopurinol and NAY groups had the effects of decreasing serum UA, CRE, and BUN levels, and especially in the medium dose group, the effect of reducing the aforementioned indexes was most significant. The excretion of UA in the hyperuricemic mice was also abnormal, and the excretion of UA, CRE, and BUN in the urine of the mice in the model group decreased significantly compared with the normal mice. NAY increased renal excretion of UA, CRE, and BUN, but did not completely normalize the abnormal excretion in hyperuricemic mice. It is inferred that NAY can also alleviate hyperuricemia by promoting renal excretion of UA, CRE, and blood BUN. Our *in vitro* experiments on AML12 and HK-2 cells show that compared with the normal group, the level of supernatant UA in the model group was significantly increased, indicating that adenosine combined with XO induced HK-2 and AML12 cells to establish the hyperuricemia cell model successfully. Both the NAY and allopurinol groups could reduce UA levels in hyperuricemia AML12 and HK-2 cell models, and the high dose group had the highest reduction. In this study, it was found that NAY had a good effect on reducing UA and improving kidney injury in mice induced by high UA without obvious damage to the liver.

Many studies showed that the rate-limiting enzymes that catalyze UA production included XOD and ADA. XOD is an existing form of xanthine oxidase (XOR) in the body and is mainly synthesized in the liver (Pang et al., 2017). It is mainly synthesized in the liver and is a key enzyme for UA production and catalyzes hypoxanthine and xanthine to eventually produce UA. Adenosine is catalyzed by ADA to produce inosine, which ultimately produces hypoxanthine (Pang et al., 2017). Therefore, it is also one of the important indicators in the study of anti-hyperuricemia. This study shows the mid-dose and high-dose groups showed significant inhibition of liver XOD activity, similar to the allopurinol group. In the AML12 cell experiment, NAY showed the effect of reducing the viability of XOD in the model group, and this effect was as potent as the positive drug allopurinol. In this study, the expression level of XOD protein in the mouse liver and AML12 cells decreased significantly under the action of the tested drug compared with the model group, which further proved that the drug inhibited the production of UA by inhibiting the activity of XOD to reduce the level of serum UA. Through molecular docking test, we can also see that NAY and XOD have certain binding ability, which further proves that NAY can inhibit the formation of UA by inhibiting XOD. These results are inconsistent with the effect of the same dose group on the serum UA level, indicating that there are other mechanisms to reduce UA, such as the role of UA transporters.

UA transporters were identified as potential therapeutic targets for hyperuricemia. Previous studies have shown that 70% of urate is mainly dependent on the renal urate transport system (Pascual and Perdiguero, 2006). The metabolic process of UA is known to include four steps of filtration reabsorption and secretion post-secretion reabsorption, with the exception of filtration, which does not require transporter participation, and the other UA metabolic processes that require urate transporters to function. Thus urate transporters can divide into two major groups: reabsorption and secretion proteins. Urate reabsorption proteins include: urate anion transporter 1 (URAT1) and glucose transporter 9 (GLUT9). Urate secretory proteins include: OAT1 and OAT3, multidrug resistance protein 4 (MRP4), and the fumarate transporter (NPT1/NPT4), which are located on the basolateral membrane. We examined the urate transporters GLUT9, OAT1, and OAT3 expression *in vitro* and *in vivo*, and the experimental results show that NAY had different regulatory effects on GLUT9, OAT1, and OAT3. As a member of the glucose transporter family, GLUT9 can accelerate the reabsorption of UA by transporting glucose (Witkowska et al., 2012). As expected, NAY could reduce the expression of GLUT9 protein in the mouse kidney and HK-2 cells. The tested drug played a role in inhibiting the reabsorption of UA. Interestingly, the results showed that the expression of OAT1 and OAT3 in the model group decreased in hyperuricemia mice and HK-2 cells, and NAY significantly inhibited this reduction. Finally, NAY was combined with the key proteins XOD, GLUT9, OAT1, and OAT3 by molecular docking, and the results showed good binding. NAY exhibited good binding with the aforementioned proteins, which also verified the results of this study to some extent. It is speculated that NAY can reduce serum UA from both promoting UA excretion and inhibiting UA reabsorption.

The NLRP3 inflammasome, a protein complex discovered in 2002 by Martinon et al. (2002), consists of NLRP3, the adapter protein ASC and the effector protein caspase-1. After the organism receives the corresponding stimulus, the NLRP3 inflammasome recruits ASC through pyd–pyd interaction, and ASC recruits the precursor Caspase-1 through card–card interaction, thereby activating Caspase-1 and regulating IL-1β and IL-18 processing and maturation, which in turn participate in the body’s inflammatory response (Schroder and Tschopp, 2010). The results showed that the protein expression levels of NLRP3, Caspase-1, and ASC were significantly increased in both renal tissues of hyperuricemia mice and adenosine-treated HK-2 cells. The levels of NLRP3 and IL-1β inflammatory factors in serum and kidney of hyperuricemia mice also increased significantly. These results suggest that the abnormal expressions of NLRP3, ASC and Caspase-1 may be related to the level of blood UA. The protein expression of NLRP3, ASC, and Caspase-1 in the kidney tissue of hyperuricemia mice and the level of mice inflammatory cytokines NLRP3 and IL-1β was significantly reduced; this change was demonstrated at each dose in the dosing groups. These results suggest that NAY has an effect on the NLRP3 signaling pathway, and the mechanism may be that the drug enters the body, reduces the formation of NLRP3 inflammasome, and blocks the signaling pathway by inhibiting the activities of NLRP3, ASC, and Caspase-1. This was further demonstrated in HK-2 cell experiments. Medium - and high-dose groups also reduced NLRP3, ASC, and Caspase-1 protein overexpression in hyperuricemia model cells. Therefore, in this study, NAY may inhibit the shearing of IL-1β precursor by blocking the activation of Caspase-1, so as to reduce the IL-1β level and have an intervention effect on the NLRP3 inflammatory signaling pathway.

## 6 Conclusion

**FIGURE 15:**
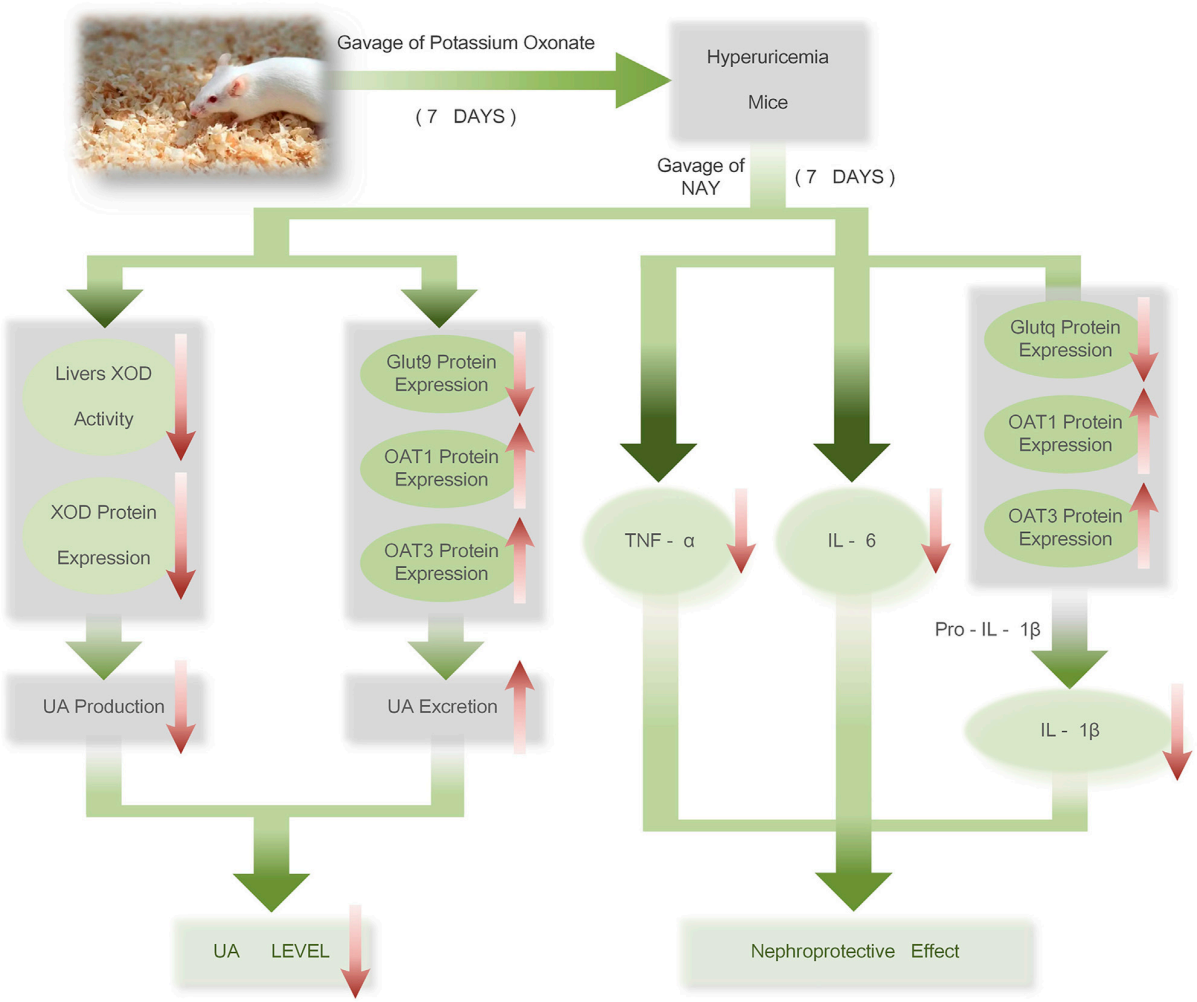
Therapeutic mechanism of NAY on hyperuricemia mice.

The therapeutic mechanism of NAY on hyperuricemia mice is shown in Figure 15. NAY reduces UA from multiple perspectives and has renal protective effect. We first evaluate the effect and mechanism of NAY-reducing UA in *in vivo* and *in vitro* experiments. The present results show that this NAY significantly inhibits XOD activity while reducing serum UA, CRE, and urea BUN levels, as well as regulating the expression of urate transporters GLUT9, OAT1, and OTA3. The NLRP3 inflammatory signaling pathway was intervened by blocking the activation of Caspase-1, inhibiting inflammatory responses. Moreover, the compounds exhibited significant protective effects against liver and kidney injury in PO-induced hyperuricemia mice, and the effect showed a little dose-dependent, the optimal dose and specific action targets of drugs need to be further studied. While having good efficacy and low toxicity ratio of the drugs, this compound has good development prospects as a future antihyperuricemia drug.

## Data Availability

The original contributions presented in the study are included in the article/Supplementary Materials; further inquiries can be directed to the corresponding authors.
